# Drought stress modifies the community structure of root-associated microbes that improve *Atractylodes lancea* growth and medicinal compound accumulation

**DOI:** 10.3389/fpls.2022.1032480

**Published:** 2022-12-02

**Authors:** Hongyang Wang, Yuefeng Wang, Chuanzhi Kang, Sheng Wang, Yan Zhang, Guang Yang, Li Zhou, Zengxu Xiang, Luqi Huang, Dahui Liu, Lanping Guo

**Affiliations:** ^1^ State Key Laboratory Breeding Base of Dao-di Herbs, National Resource Center for Chinese Materia Medica, China Academy of Chinese Medical Sciences, Beijing, China; ^2^ Key Laboratory of Biology and Cultivation of Herb Medicine, Ministry of Agriculture and Rural Affairs, Beijing, China; ^3^ College of Horticulture, Nanjing Agricultural University, Nanjing, China; ^4^ Chinese Academy of Chinese Medical Sciences, Beijing, China; ^5^ Pharmacy Faculty, Hubei University of Chinese Medicine, Wuhan, China

**Keywords:** *A. lancea*, drought stress, medicinal compounds, rhizosphere microbe, endophytic microbe

## Abstract

*Atractylodes lancea* is an important medicinal plant in traditional Chinese medicine, its rhizome is rich of volatile secondary metabolites with medicinal values and is largely demanded in modern markets. Currently, supply of high-yield, high-quality *A. lancea* is mainly achieved *via* cultivation. Certain soil microbes can benefit plant growth, secondary metabolism and induce resistance to environmental stresses. Hence, studies on the effects of soil microbe communities and isolates microorganisms on *A. lancea* is extremely meaningful for future application of microbes on cultivation. Here we investigated the effects of the inoculation with an entire soil microbial community on the growth, resistance to drought, and accumulation of major medicinal compounds (hinesol, β-eudesmol, atractylon and atractylodin) of *A. lancea*. We analyzed the interaction between *A. lancea* and the soil microbes at the phylum and genus levels under drought stress of different severities (inflicted by 0%, 10% and 25% PEG6000 treatments). Our results showed that inoculation with soil microbes promoted the growth, root biomass yield, medicinal compound accumulation, and rendered drought-resistant traits of *A. lancea*, including relatively high root:shoot ratio and high root water content under drought. Moreover, our results suggested drought stress was more powerful than the selectivity of *A. lancea* in shaping the root-associated microbial communities; also, the fungal communities had a stronger role than the bacterial communities in protecting *A. lancea* from drought. Specific microbial clades that might have a role in protecting *A. lancea* from drought stress were identified: at the genus level, the rhizospheric bacteria *Bacillus*, *Dylla* and *Actinomadura*, and rhizospheric fungi *Chaetomium*, *Acrophialophora*, *Trichoderma* and *Thielava*, the root endophytic bacteria *Burkholderia-Caballeronia-Paraburkholderia*, *Allorhizobium-Neorhizobium-Pararhizobium-Rhizobium*, *Dylla* and *Actinomadura*, and the root endophytic fungus *Fusarium* were closely associated with *A. lancea* under drought stress. Additionally, we acquired several endophytic *Paenibacillus*, *Paraburkholderia* and *Fusarium* strains and verified they had differential promoting effects on the medicinal compound accumulation in *A. lancea* root. This study reports the interaction between *A. lancea* and soil microbe communities under drought stress, and provides insights for improving the outcomes in *A. lancea* farming *via* applying microbe inoculation.

## Introduction

*Atractylodes lancea* is a traditional Chinese medicinal herb that is widely distributed in China, Japan and the Korean peninsula. Its medicinal properties are well known across East Asia (Tsusaka et al., 2019). In China the rhizome of the perennial herb *A. lancea* has been used to treat digestive disorders, night blindness, influenza and rheumatic diseases since ancient times (Wang et al., 2008; Nie, 2018; Qu et al., 2018). The *A. lancea* rhizome is particularly rich in specific volatile organic compounds, sesquiterpenoids. These are characteristic secondary metabolites that endow the *A. lancea* rhizome with high pharmaceutical value (Guo et al., 2002). Among the volatile compounds that are enriched in the rhizome, four compounds, the volatile sesquiterpenoids hinesol, β-eudesmol, atractylon and the polyacetylene-type atractylodin are currently approved by the Pharmacopoeia of the People’s Republic of China as major medicinal compounds. Their concentration determines the quality and pharmaceutical value of *A. lancea* samples (Committee., 2020). Modern medical researche has uncovered multiple novel functions of these four compounds. For examples, hinesol can exert antitumor effects (Guo et al., 2019); β-eudesmol exerts antitumor and antiangiogenic activity and can act as a chemosensitizing agent in therapies for drug-resistant cancers (Tsuneki et al., 2005; Ma et al., 2008; Acharya et al., 2021); atractylon has antihypertensive, anti-aging and anti-inflammatory effects (Cheng et al., 2022); atractylodin exhibits antibacterial, anti-inflammatory and hepatoprotective activities (Lyu et al., 2019). Its long history in traditional medicine coupled with modern research findings have created high demand for high-quality *A. lancea* in the Chinese and East Asian markets.

Chines medical tradition often singles out particular geographic locations where the highest quality exemplars of a plant grow. These are known as “Dao-di” (Geo-authentic) herbs. The Geo-authentic *A. lancea* contains high concentrations of sesquiterpenoid secondary metabolites, especially hinesol, β-eudesmol, atractylon and atractylodin. It is distributed in the mountainous areas of Jiangsu Province in China (approximately 30°45′32′′ to 35°7′15′′ N, 116°21′28′′ to ′′121°56′38′′ W). Currently, wild Geo-authentic *A. lancea* resources are gravely endangered (Zhou et al., 2016). Therefore, farming is the major source of high-quality *A. lancea* that can meet the demands of the international market. However, as a perennial medicinal plant, *A. lancea* is vulnerable to soil-borne disease outbreaks due to monocropping (Wang et al., 2016). Furthermore, its pharmaceutical value becomes degraded when the balance between the primary and secondary metabolism is disturbed when it is cultivated on open farms and managed with common fertilization regimes. There is an urgent need to improve farming strategies for *A. lancea*. For plants, up-regulation of the biosynthesis of certain groups of secondary metabolites is an important strategy, sometimes an inevitable outcome, of their response and adaptation to environmental changes, stresses and microbe induction or infection (Oh et al., 2009; Meraj et al., 2020). We have carried out a series of studies on the environmental factors that impact Geo-authentic *A. lancea* quality, in terms of rhizome accumulation of secondary metabolites and medicinal compounds. The results show that development of high-quality Geo-authentic *A. lancea* is dependent on particular local climate conditions and local wild soil microbe communities (unpublished data). Soil microbe community composition is amenable to modification and, given further research, could prove the optimal solution to improving the quality of farmed *A. lancea.*

Soil microbes play a vital role in regulating the growth, development, stress resistance and metabolism of plants (Jacoby et al., 2017; Abdel Motaleb et al., 2020; Devanathan et al., 2021; Pang et al., 2021). The impact of microbes on plants is highly varied: certain soil microbes can be pathogenic, while others can be beneficial (Berg et al., 2020). Remarkably, soil microbes can impact the secondary metabolism of plants, usually *via* exerting promoting effects. This is closely associated with the pharmaceutical value of medicinal plants, because the medicinal compounds are mostly, if not all, plant secondary metabolites (Bednarek and Osbourn, 2009; Chen et al., 2018; Korenblum and Aharoni, 2019). For examples, inoculation with endophytic *Trichoderma citrinoviride* ginseng (could) promote the biosynthesis of ginsenosides (Park et al., 2019). Inoculation with combination of particular strains of bacteria could improve the accumulation of astragaloside IV and calycosin-7-glucoside in the root of *Astragalus mongholicus* (Lin et al., 2022). Inoculation with *Glomus mosseae* promoted the concentration of glycyrrhizic acid, liquiritin, isoliquiritin, and isoliquiritigenin in the main root of liquorice (*Glycyrrhiza uralensis*) (Chen et al., 2017). Therefore, studying the impacts of soil microbes on the secondary metabolite accumulation and stress tolerance of medicinal plants is crucial to elucidate the role soil microbes play in determining herb quality. Furthermore, such studies would help clarify the effects of inoculation with certain microbe mixtures, clades or isolates microorganisms on the quality of the host medicinal herbs cultured on farms. Beneficial microbes have been applied in agriculture and proven highly effective for improving crop production and disease resistance (Kumar et al., 2016; Afridi et al., 2022; Ali et al., 2022). Collectively, to apply microbes on cultivated *A. lancea* is a potentially powerful solution to the current difficulties faced by *A. lancea* farming. Indeed, in a previous study, we found that the local soil microbes of the Geo-authentic *A. lancea* habitat could promote the accumulation of the four major medicinal compounds in the *A. lancea* root while rendering the *A. lancea* plantlets tolerant to heat stress (Wang et al., 2022). In the current study, we investigated the impact of the local soil microbes from a Geo-authentic *A. lancea* habitat on the fitness and medicinal compound accumulation of *A. lancea* under water deficiency stress, and how *A. lancea* and the soil microbes interacted symbiotically.

Drought stress can promote production of terpenoids or sesquiterpenoids in aromatic plants. For examples, the production of terpenoids in the root of the aromatic plant *Tanacetum vulgare* L. is significantly promoted under both drought and herbivory stresses. Moreover, the root of *T*. *vulgare* L. is rich in sesquiterpenoids with their concentration increasing further under drought conditions (Kleine and Müller, 2014). Llorens-Molina and Vacas (2017) report that for certain chemotypes (population) of the aromatic plant thyme (*Thymus vulgaris* L.) in Spain, the composition of thyme volatile oil is different between May and August, an annual period of drought in Mediterranean climates. Particularly, the proportion of sesquiterpenoids in the volatile oil of the population Navarrete increased about fourfold from May to August. In sage (*Salvia officinalis*), drought stress can strongly induce the expression of multiple monoterpene synthase genes and promote the accumulation of monoterpenes (Radwan et al., 2017). Furthermore, certain microbe strains have been implicated in the regulation of medicinal sesquiterpenoid metabolism in *A. lancea* and other plants. For examples, Christensen et al. (2018) report production of a sesquiterpene keto acid derivative of β-macrocarpene, zealexin A4, with antimicrobial activity in maize. Production of zealexin A4 was elevated when subjected to either fungal pathogen infection or herbivore damage. The inoculation with an endophytic *Pseudomonas fluorescens* strain altered the metabolic flux in *A. lancea* and specifically enhanced sesquiterpenoid accumulation (Zhou et al., 2018). The endophytic fungus *Gilmaniella* sp. AL12 induced ethylene production in *A. lancea* and thus enhanced sesquiterpenoid accumulation *via* ethylene signaling (Yuan et al., 2016). Taken together, these findings offer the tantalizing possibility of manipulating *A. lancea* growth and medicinal compound accumulation by adjusting soil microbe communities under drought stress. Moreover, it would be of great theoretical and practical importance to ascertain the functions exerted by particular soil microbe clades or isolates microorganisms on *A. lancea* medicinal compound accumulation. The present study aims to advance our knowledge in this area.

We used polyethylene glycol 6000 (PEG6000) to mimic drought stress and performed inoculation assays with the soil microbe mixture from a Geo-authentic *A. lancea* habitat under controlled nursery conditions. We performed comparative analyses on the phenotypes of the *A. lancea* plantlets post-inoculation, and on the soil microbe community structure under multiple drought scenarios we established. Moreover, we isolated endophyte microbe strains with application potentials and verified their function on *A. lancea* medicinal compound accumulation.

## Materials and methods

### Plant material and soil microbe inoculum

Seeds of wild Geo-authentic *A. lancea* were collected at Jin-Niu-Dong-Shan (Mount ‘Jin-Niu-Dong’, 31°46′37″ N, 119°18′52″ W), Jintan City, Jiangsu Province. Surface-sterilized seeds were placed on Murashige & Skoog (MS) medium to germinate surface-sterile plantlets. The aerial part of the approximately 2-3 cm tall plantlets were cut and cultured on solid MS medium (pH = 5.8) containing 30 g/L sucrose, 0.1 mg/L naphthalene acetic acid (NAA) and 1 mg/L 6-benzyladenine (6-BA) for vegetative propagation *via* tillering. Rooting was performed by culturing four-week-old vegetatively propagated *A. lancea* plantlets (approximately 4-cm tall) on the rooting medium, which was solid MS with 30 g/L sucrose and 0.5 mg/L NAA, for another four weeks.

Geo-authentic soil was collected approximately 5-10 cm beneath the surface at five random sites in a forest-covered mountainous area (31° 36′ 18″ N, 119° 6′ 48″ E) in Lishui District, Nanjing City, Jiangsu Province. The Geo-authentic soil samples were then mixed thoroughly for further use. The water suspension of the mixed Geo-authentic soil sample, which contained an entire Geo-authentic microbial community, was used as the soil microbe inoculum. 10 g of the mixed Geo-authentic soil sample was placed in 100 mL of sterile water, then oscillated on a shaker at 220 revolutions per minute (rpm) for 10 minutes (min) to produce the soil microbe inoculum. The soil microbe inoculum that was autoclaved for 1 h at 121 °C was used as the mock inoculum.

### Drought treatment and inoculation

Plantlets with approximately 2-cm-long adventitious roots were carefully harvested from the rooting medium and planted in the sterile mixture (hereafter referred to as ‘soil’) of peat soil (Jiffy product, Netherlands) and vermiculite (6:1, v/v) under sterile conditions. For different drought treatments, soil mixture was pre-mixed with 0%, 10% and 25% PEG6000 solutions (w:v) to simulate no drought, mild drought and severe drought contexts, respectively, as described by (He et al., 2022). The ratio of PEG6000 solution to the soil mixture was 1:1.2 (w:w). The *A. lancea* plantlets were then placed in a plant nursery room set at approximately room temperature (23 ± 2°C, referred to as ‘room temperature’) and with a 12 hour (h)/12 h light/dark cycle for nine days before soil microbe inoculation. For each treatment, 30 biological replicates were prepared as a group.

**Figure 1:**
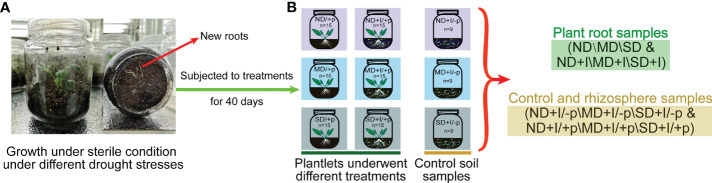
Schematic diagrams of the workflow of this study. **(A)** The drought stress and microbial inoculation treatments. **(B)** Samples subjected to microbial diversity analyses. ND, no drought stress (0% PEG6000); MD, mild drought stress (10% PEG6000); SD, severe drought stress (25% PEG6000); +I, with Geo-authentic soil microbe inoculation; +p, with *A lancea* plantlet; -p, without *A ancea* plantlet.

After 9 days of growth, new roots could be observed at the bottom of the glass bottle, indicating that the plantlets had successfully rooted and survived in the soil (**Figure 1**). Subsequently, 15 plantlets were randomly selected from each group to be inoculated with 5 mL of soil microbe inoculum; the other 15 plantlets of the group were inoculated with the mock inoculum under sterile conditions. In addition, bottled soil samples without *A. lancea* plantlets were also prepared and inoculated to be used as control samples (**Figure 1**) with 9 replicates prepared for each group. Every three replicates were mixed into one biological replicate for soil microbial sequencing. All plantlets were then cultured in the plant nursery for another 30 days.

### Sample collection and measurement of biomass

At 40-day old, *A. lancea* plantlets had developed multiple adventitious roots and barely recognizable rhizomes. The compartmentalization between rhizome and adventitious root was unclear. Hence, in this study we simply referred to the underground compartment of the 40-day-old *A. lancea* plantlets as the root, while the aerial compartment was referred to as the shoot. Firstly, rhizosphere soil samples of all the 15 A*. lancea* plantlets were collected. The plantlets were very carefully removed from the soil to avoid breaking and loss of root. The soil remaining on the root surface was gently removed. The thin soil that appeared to be adhering to the root surface was then carefully removed and collected as our rhizosphere soil samples; we believe it was the soil within 1 mm from the root. The rhizosphere soil samples were stored in liquid nitrogen immediately after collection. Due to the scarcity of rhizosphere soil samples, all the plantlets were used for sample collection. Five random samples of each experimental group were pooled as one biological replicate, resulting in three biological replicates in total. The plantlets were then all rinsed using sterile distilled water and six plantlets were randomly selected from each treatment group for the measurement of biomass. They were subsequently also used for the measurement of volatile compounds.

Dry weight data was measured after freeze-drying for approximately 72 h to constant weight. We performed freeze-drying instead of heat-drying to maintain the volatile compounds in the dry root samples for subsequent measurements. The root samples of two individual plantlets were pooled as one biological replicate, resulting in three biological replicates in total. The remaining nine plantlets of each group were collected and three individual plantlets were pooled as one biological replicate, resulting in three biological replicates for the root endophyte analyses. The samples used for endophyte analyses were placed in clean 50 mL conical tubes and pre-rinsed three times with sterile distilled water. The washed roots were then treated with 70% ethanol for 10 min, followed by treatment with 2.5% sodium hypochlorite and sonication for an additional 10 min. The samples were then drained and rinsed with sterile distilled water three times. To check for surface sterility, 100 μL of the final rinsed solution was plated in Potato Dextrose Agar (PDA) and Nutrient agar (NA) resulting in zero colonies.

**Figure 2:**
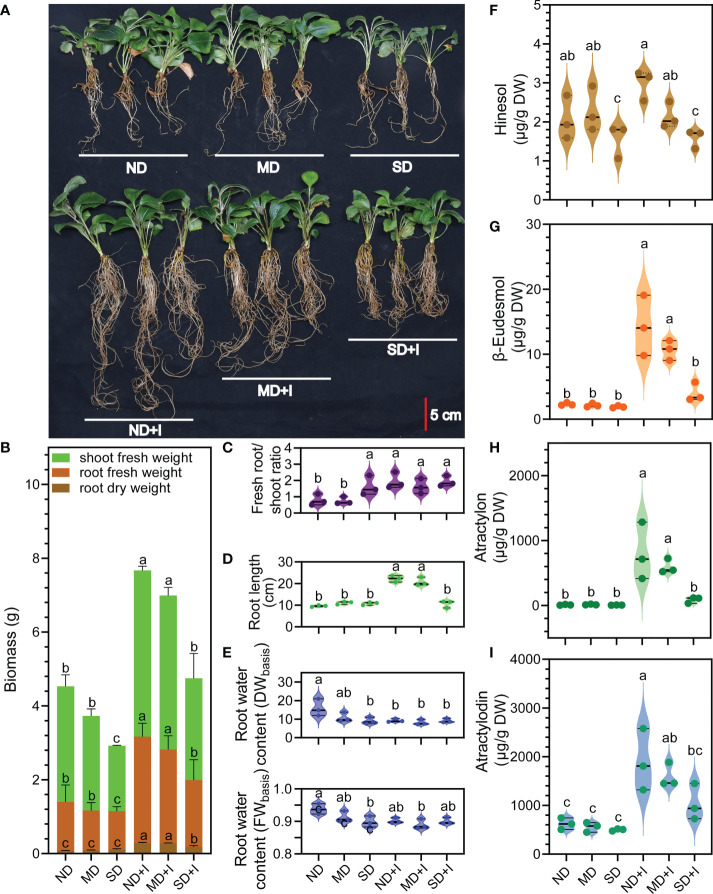
Phenotype and concentration of four major medicinal compounds of A lancea under different treatments conditions. **(A)** Phenotype. **(B–I)**: n = 3. Data were shown as mean ± SD. Different lower-case letters represent significant differences (one-way ANOVA, *P* < 0.05). ND, no drought stress (0% PEG6000); MD, mild drought stress (10% PEG6000); SD, severe drought stress (25% PEG6000); +I, with Geo-authentic soil microbe inoculation; DW, dry weight; FW, fresh weight.

According to Turner (1981) the water content (WC) of plant tissues can be expressed on either a dry weight (DW) or a fresh weight (FW) basis. The formulas were: WC_(DW basis)_ = (FW – DW)/DW × 100 and WC_(FW basis)_ = (FW – DW)/FW × 100. However, the results calculated *via* these formulas could be influenced by plant growth and DW accumulation through the duration of the experiments. Hence, an alternative formula involving the turgid weight (TW), which is the most widely applied formula, was proposed for calculating the relative water content (RWC): RWC = (FW – DW)/(TW – DW) × 100. In this study, the comparison of water content among our materials does not involve changes in DW because our measurement of biomass was performed only once at the same time for all the samples. Moreover, since our samples were used for subsequent measurement of the volatile compound concentration data, we did not put our samples through treatments to reach full turgor and did not measure the TW in order to avoid impacts of such treatments on the volatile compound concentration. In this study the water content of *A. lancea* root was calculated on both the DW and FW bases *via* the formulas WC_(DW basis)_ = (FW – DW)/DW and WC_(FW basis)_ = (FW – DW)/FW (**Figure 2E**).

### Measurement of volatile medicinal compounds

Freeze-dried root samples were ground into fine powder. Approximately 0.1 g of the powder was carefully measured placed in an Eppendorf tube, then 400 μL of analytically pure n-hexane was added to the powder and mixed thoroughly. Extraction was performed *via* ultrasound treatment at 60 Hz for 15 min. The mixture was then centrifuged at 5000 × g and 4°C for 5 min. The supernatant was filtered through 0.22-μm PES membrane filter capsules (Sterivex; Millipore) and subjected to GC-MS analysis. The concentration of hinesol, β-eudesmol, atractylon, and atractylodin in freeze-dried root samples was measured *via* gas chromatography coupled with mass spectrometry (GC-MS) using a Trace 1310 series GC with a TSQ8000 MS detector (Thermo Fisher Scientific Co. Ltd, Waltham, Massachusetts, USA) and a TR-5ms capillary column (30 m 3 0.25 mm i.d., DF = 0.25 mm, Thermo Fisher Scientific). Following Vannier et al. (2018) with slight modifications the injected sample (1 μL) was separated at the Helium flow rate of 1 mL/min; the temperature program was 2 min at 120°C followed by a gradient from 120°C to 240°C at 5°C/min, and held at 240°C for 5 min; the injector and detector temperatures were set at 240°C and 350°C, respectively. The MS operating conditions were as follows: the MS ionization mode indicated the electron impact ion source (EI) at 230°C, with an acceleration voltage of 70 eV. The interface temperature was 240°C and the total ion current was recorded for a mass range of 40–500 amu. The contents of four volatile oils in each sample were quantitatively determined by the standard curves (see **Supplementary Table 1**).

### Endophytic and rhizosphere microbial community analyses

**Figure 1B** showed the plant and soil samples that were tested for microbe communities and the subsequent work flow. The total microbial DNA for 16S and ITS amplicon sequencing was extracted from 100 mg of *A. lancea* root sample or 150 mg of soil sample using the PowerSoil DNA Isolation Kit (Mo Bio Laboratories, Solana Beach, CA, USA) per the manufacturer’s instructions. The quality of the extracted DNA was verified *via* 1% agarose gel electrophoresis. Primers 799F (5´-AACMGGATTAGATACCCKG-3´) (Bulgarelli et al., 2012) - 1392R (5´-ACGGGCGGTGTGTRC-3´) (Lundberg et al., 2012) and 799F (5´-AACMGGATTAGATACCCKG-3´) - 1193R (5´-ACGTCATCCCCACCTTCC-3´) (Bulgarelli et al., 2015) amplified the variable V5-V7 region of 16S rRNA in two steps. Primer ITS3F (5’-GCATCGATGAAGAACGCA GC-3’) - ITS4R (5’-TCCTCCGCTTATTGATATGC-3’) (Toju et al., 2012) amplified ITS2 region. Both forward primers and reverse primers were added with error correction barcode (Hamady et al., 2008).

The amplification was carried out *via* PCR using a GeneAmp 9700 PCR system (Applied Biosystems, Foster City, CA, USA). The total reaction volume was 25 μL, including 1 μL DNA template, 0.5 μL forward primer, 0.5 μL reverse primer, 0.25 μL bovine serum albumin, 12.5 μL 2 × DreamTaq Green PCR Master Mix (Thermo Scientific, USA), replenished with ddH_2_O to 25 μL. Setting three technical replicates for each reaction, PCR was carried out as follows: 95°C for 3 min, followed by 27 cycles of 95°C for 30 s, 55°C for 30 s, and 72°C for 45 s, and a final extension at 72°C for 10 min. Three technical repeats of one sample were mixed into a single PCR product. The products were separated *via* 2% agarose gel electrophoresis and purified using a Qiagen PCR purification ki5t (Qiagen, Hilden, Germany). Furthermore, the purified products were quantified with Pico Green using a QuantiFluorTM-ST Fluorometer (Promega Biotech, Beijing, China) and were then pooled at equal concentrations. Thereafter, the amplicons were sequenced in an Illumina MiSeq platform (San Diego, CA, USA) at Shanghai Majorbio Bio-pharm Technology Co., Ltd., Shanghai, China.

The data were analyzed on the online platform Majorbio Cloud Platform (www.majorbio.com) (data had been uploaded to NCBI, project number PRJNA806199). Paired-end (PE) reads (average length 376 bp and 341 bp) obtained by MiSeq sequencing were spliced according to their overlap relations using FLASH (Magoč and Salzberg, 2011). Quality control was performed *via* filtering using Trimmomatic (Bolger et al., 2014). All sequences were clustered into operational taxonomic units (OTUs) with 97% similarity or greater using UPARSE (version 7.0) (Edgar, 2013), and a majority consensus taxonomy was obtained for each OTU. Singletons were removed from the datasets to minimize the impact of sequencing artifacts (Dickie, 2010). Chimeric sequences were identified and removed using UCHIME (Edgar et al., 2011). In order to obtain the species classification information corresponding to each OTU, the RDP classifier algorithm (https://sourceforge.net/projects/rdp-classifier/) was applied to compare the OTU representative sequences with the Silva database (Release138, http://www.arb-silva.de) and Unite*(Release 8.0, http://unite.ut.ee/index.php)for taxonomic analysis using 97% confidence threshold. Among these, chloroplasts and mitochondrial sequences were removed. The bacterial and fungal community diversity and richness were demonstrated using the Shannon and Chao1 indexes using Mothur v.1.30.1 (Schloss et al., 2009). The relative abundance bar of bacteria and fungi at the phylum level was visualized using R language tools (v.3.3.1). Principal Coordinates Analysis (PCoA) analyses was performed using QIIME (version 1.9.1) based on unweighted UniFrac distance matrix or Bray-Curtis dissimilarity. Linear discriminant analysis (LDA) coupled with effect size measurements (LEfSe) analysis was completed on the online website Galaxy (http://huttenhower.sph.harvard.edu/galaxy), in which the threshold of LDA discriminant analysis of rhizosphere bacteria was 4, and the threshold of LDA discriminant analysis of rhizosphere fungi, bacteria and fungi in roots was 2. The Student’s *t*-test within STAMP (Parks et al., 2014) was used to identify the phyla, genera and OTUs that showed significant differences in abundance between groups (confidence interval method). Psych packages were used to calculate Pearson’s rank correlation and *q* value (Revelle, 2017), and the phatmap package in R (v.3.3.1) was used to visualize the results (Kolde and Kolde, 2018).

### Verification of root endophyte function

We manually isolated multiple endophyte stains (both bacteria and fungi) from the underground compartments of wild Geo-authentic *A. lancea* plants *via* subculturing using different types of microbe-culturing media, and preserved the isolates microorganisms in-house. The endophytic *Paenibacillus* and *Paraburkholderia* strains used in this study were cultured using trypticase soy medium by oscillating at 30°C and 220 rpm for 48 hours. Then, the culture was centrifuged at 4°C and 3,000 rpm for 5 minutes to collect the bacteria. The collected bacteria were then resuspended in potato dextrose medium to reach a final concentration of 1×10^8^ cfu to be used as the inoculum. The endophytic eFungus_1, eFungus_2, eFungus_3, eFungus_4 and *Fusarium solani* were cultured using potato dextrose medium by oscillating at 30°C and 220 rpm for seven days. The culture was then filtered using autoclaved multiple-layer gauze to remove bulk mycelia under sterile conditions. The filtered culture was then centrifuged at 4°C and 3,000 rpm for 5 minutes to collect conidia. The collected conidia were resuspended in potato dextrose medium to reach a final concentration of 1×10^8^ cfu to be used as the inoculum. Autoclaved potato dextrose medium was used as the mock inoculum. *A. lancea* plantlets that were transplanted in autoclaved bottled soil under sterile conditions and had grown for nine days with visible new adventitious roots were used for the inoculation assays. Each plantlet was inoculated with 5 mL of inoculum under sterile conditions, then grown for 40 days before collected for measurement of medicinal compound in the root.

### Statistical analysis

Data were recorded and processed by Excel (Office 2019). GraphPad Prism 8.0.1 (GraphPad Software Inc., USA) was used for rendering graphics. One-way ANOVA was performed using IBM SPSS Statistics 19.0 (SPSS, Chicago, IL, USA). Results were expressed as mean ± standard deviation (S.D.).

## Results

### Soil microbe inoculation promoted the growth and major medicinal compound accumulation of *A. lancea* under drought stress

The results showed that GSM inoculation greatly altered the root architecture of the *A. lancea* plantlets: the number and length of the adventitious roots evidently increased while induced by GSM (**Figures 2A, D**), while the aerial/root fresh biomass, root dry mass, and root water content were significantly improved (**Figures 2B, E**). Notably, the root:shoot ratio of uninoculated *A. lancea* significantly increased under 25% PEG6000 treatment compared to 0% or 10% PEG6000 treatment (**Figure 2C**), representing a drought-resistant trait (Chen et al., 2022). *A. lancea* plantlets inoculated with GSM acquired significantly higher root:shoot ratio compared to the uninoculated plantlets, indicating improved resilience against water deficiency stress.

The inoculation with GSM significantly improved the concentration of all the four major medicinal compounds in *A. lancea* root under normal growth conditions (**Figures 2F–I**). Under 10% PEG6000 treatment, GSM inoculation significantly increased accumulation of β-eudesmol, atractylon and atractylodin. However, under 25% PEG6000 treatment, GSM inoculation was only able to induce a slight increases in atractylodin accumulation (not to a statistically significant extent). Thus, the accumulation of medicinal compounds was impaired under 25% PEG6000 treatment.

### Changes in the bacterial and fungal community composition were distinct in the rhizosphere of *A. lancea* under different PEG6000 treatments

Since the growth traits and major medicinal compound accumulation of *A. lancea* were significantly altered by GSM induction, we investigated the changes of the rhizosphere microbe communities pre- and post- inoculation. We performed 16S rRNA and ITS amplicon sequencing to reveal the bacterial and fungal communities, respectively, in the rhizosphere of the *A. lancea* plantlets under different treatment conditions. A total of 1.80 million high-quality 16S rRNA sequence tags with an average length of 376 bp, and 3.03 million high-quality ITS sequence tags with an average length of 343 bp were generated for all the sequenced *A. lancea* rhizosphere soil samples and the bottled soil samples without *A. lancea* plantlets. Subsequently, 767 OTUs of the rhizospheric bacteria corresponding to 19 phyla and 293 genera, and 286 OTUs of the rhizospheric fungi corresponding to 9 phyla and 137 genera were obtained and annotated.

**Figure 3:**
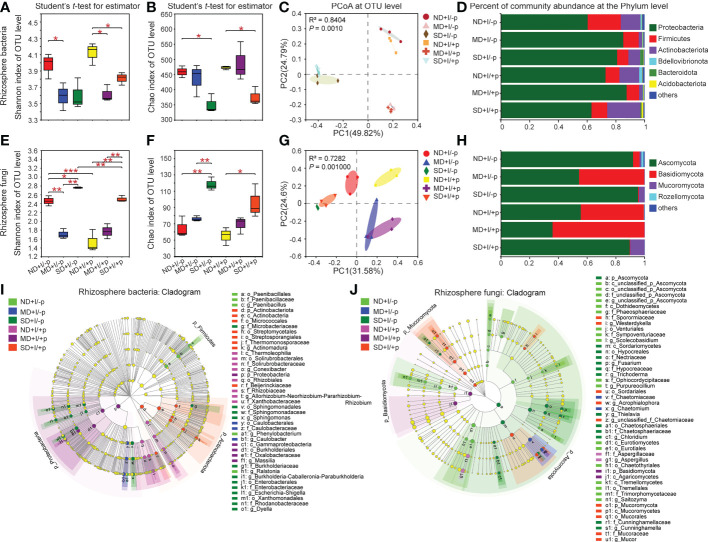
Diversity and community structure of rhizosphere bacteria and fungi of *A. lancea*. Shannon index **(A, E)** shows α diversity, Chao1 index **(B, F)** shows richness, both at the OTU level. C and G: PCoA of rhizosphere bacteria **(C)** and Fungi **(G)** in *A. lancea* based on Bray-Curtis matrix at the OTU level (n = 3). D and H: Bar chart of rhizosphere bacteria **(D)** and fungi **(H)** in *A. lancea* rhizosphere at phylum level. **(I, J)** Cladogram showing the phylogenetic distribution of the bacterial (**I**, LDA score = 4) and fungal (**J**, LDA score = 2) lineages associated with the bottled soil and rhizosphere soil of *A. lancea*. Circles indicate phylogenetic level from phylum to genus. The diameter of each circle is proportional to the abundance of the group. Asterisks represent significant difference by Student’s t-test: *P < 0.05; **P < 0.01; ***P < 0.001; ND, no drought stress, 0% PEG6000; MD, mild drought stress, 10% PEG6000; SD, severe drought stress, 25% PEG6000; +I, with Geo-authentic soil microbe inoculation; +p, with *A. lancea* plantlet.

We performed comparative analyses on the OTUs, and the results showed that PEG6000 treatment had a significant impact on the diversity and richness of the microbial communities in the rhizosphere of *A. lancea*. The Shannon index (**Figure 3A**) and Chao1 index (**Figure 3B**) revealed lower diversity and richness of the bacteria in *A. lancea* rhizosphere and the bottled soil under PEG6000 treatment compared to normal growth conditions, with or without the *A. lancea* plantlets. Notably, in both the rhizosphere and the bottled soil, the decrease in the diversity of bacteria appeared more susceptible to PEG6000 treatment: bacterial diversity significantly decreased under both 10% and 25% PEG6000 treatment (**Figure 3A**) whereas bacterial richness only decreased when the concentration of PEG6000 reached 25% (**Figure 3B**). Results of the principal coordinate analyses (PCoA) based on the Bray-Curtis distance algorithm at the OTU level showed remarkable similarity in the bacterial community composition between the rhizosphere soil of *A. lancea* and the soil without *A. lancea* plantlets post-GSM-inoculation under each treatment (0%, 10% or 25% PEG6000), suggesting that the concentration of PEG6000 had the greater impact on the bacterial community composition in the soil than proximity to the *A. lancea* (**Figure 3C**).

As for the rhizosphere fungal community composition of *A. lancea*, in contrast to that of the bacteria, the Shannon index (**Figure 3E**) and Chao1 index (**Figure 3F**) demonstrated increased fungal diversity and richness under PEG6000 treatment with positive correlation to the PEG6000 concentration (**Supplementary Data 1**) in both the *A. lancea* rhizosphere and the bottled soil. The PCoA for the fungal communities at the OTU level revealed the relatively high similarity of the fungal community composition under 25% or 10% PEG6000 treatment between the *A. lancea* rhizosphere soil and the bottled soil post-GSM-inoculation. However, when there was no PEG6000 treatment, the fungal communities appeared distinguished between the rhizosphere of *A. lancea* and the bottled soil without *A. lancea* plantlets (by PC1, as shown in **Figure 3G**). Curiously, the diversity of the fungal communities was remarkably high in the GSM-inoculated bottled soil alone, breaking the pattern of fungal community diversity being positively correlated to the PEG6000 concentration (**Figures 3E, F**), suggesting the significant impact and strong selectivity of *A. lancea* on the soil fungal communities in its vicinity.

### *A. lancea* differentially enriched and excluded particular clades of microbes in its rhizosphere under varying drought contexts

Considerable specificity was identified in the bacterial community composition of the *A. lancea* rhizosphere. At the phylum level (**Figure 3D**; **Supplementary Figures 1A–C**), Proteobacteria (*P* = 0.0526) and Bdellovibrionota (*P* = 0.0155) were specifically enriched in *A. lancea* rhizosphere post-GSM-inoculation under 0% PEG6000 treatment compared to the bottled soil inoculated with GSM. But Actinobacteriota (*P* = 0.0450) and Acidobacteriota (*P* = 0.0017) were significantly enriched, while the relative abundance of Proteobacteria (*P* = 0.0520) was reduced under 25% PEG6000 treatment compared to the bottled soil inoculated with GSM. At the genus level (**Supplementary Figures 1D–F**), *Allorhizobium-Neorhizobium-Pararhizobium-Rhizobium* (hereinafter referred to as *A-N-P-R*) appeared enriched in the rhizosphere of *A. lancea* under different PEG6000 treatment (0%, *P* = 0.0252; 10%, *P* = 0.0238; 25%, *P* = 0.4703) compared to the bottled soil post-GSM-inoculation. These specialized bacterial communities might have a role in protecting *A. lancea* under water deficiency stress.

A cladogram displaying these bacterial communities at multiple clades was generated using the LEfSe tool. Groups of bacteria with the LDA score = 4 were identified as significantly enriched (**Figure 3I**). This tool allows analyzing microbial community data at any clade. In the rhizosphere of *A. lancea* under 0% PEG6000 treatment (ND+I/+p), three groups of bacteria were significantly enriched: *Conexibacter* (from class to genus), *A-N-P-R* (from order to genus) and Xanthobacteraceae (from order to family). In the rhizosphere of *A. lancea* under 10% PEG6000 treatment (MD+I/+p), one group of bacteria, *Massilia* (from phylum to genus), was significantly enriched. In the rhizosphere of *A. lancea* under 25% PEG6000 treatment (SD+I/+p), three groups of bacteria were significantly enriched: *Actinomadura* (from phylum to genus), Streptomycetales (from phylum to order) and Micrococcales (from phylum to order).

Comparative analyses of the fungal groups revealed that at the phylum level, the relative abundance of the fungal phyla Ascomycota and the genera *Chaetomium* (*P* = 0.0578), *Talaromyces*, *Acrophialophora* and *Saitozyma* decreased, while the relative abundance of the phylum Basidiomycota and the genus *Sebacinales* (*P* = 0.0625) increased under 0% PEG6000 treatment in *A. lancea* rhizosphere compared to bottled soil post-GSM-inoculation (**Supplementary Figure 2**). Under 10% PEG6000 treatment, *A. lancea* specifically excluded the genera *Chaetomium*, *Acrophialophora* and *Thielavia*. Under 25% PEG6000 treatment, *A. lancea* specifically enriched the genus *Talaromyces* and excluded the genera *Sordaria* and *Chloridium* (**Supplementary Figure 2**). Comparing the fungal communities of the bottled soil incubated with different PEG6000 treatments without the *A. lancea* plantlets, at the genus level, *Aspergillus*, *Talaromyces*, *Neocosmospora*, *Saitozyma* appeared drought-sensitive under 10% PEG treatment since their relative abundance decreased; while under 25% PEG treatment, *Fusarium*, *Neocosmospora* (*P* = 0.0559), *Trichoderma*, *Sordaria* and *Chloridium* appeared drought-resistant, *Aspergillus*, *Talaromyces* and *Saitozyma* appeared drought-sensitive (**Supplementary Figure 8**). Comparing the fungal communities in the rhizosphere of GSM-inoculated *A. lancea* undergone different PEG6000 treatments, the genera *Chaetomium* and *Acrophialophora* were enriched and *Aspergillus* was depleted specifically under 10% PEG6000 treatment; under 25% PEG6000 treatment, the genera *Chaetomium*, *Acrophialophora*, *Trichoderma* and *Thielava* were enriched in the rhizosphere, while the genus *Aspergillus* was depleted, consistent with the drought-sensitivity of *Aspergillus* that we found (**Supplementary Figure 8**).

The cladogram of the fungal communities (**Figure 3J**) showed that fungi from phylum to genus were rarely enriched in the rhizosphere of *A. lancea* under different PEG6000 concentration. In the rhizosphere of *A. lancea* under 0% PEG6000 treatment (ND+I/+p), only one group of fungi was significantly enriched, namely *Aspergillus* (from family to genus). Similarly, under 10% PEG6000 treatment (MD+I/+p), only one group of fungi, *Agaricomycetes*, was significantly enriched (from phylum to order). Under 25% PEG6000 treatment (SD+I/+p), four groups of bacteria were significantly enriched, namely *Mucor* (from phylum to genus), *unclassified_f_Chaetomiaceae* (from order to genus), *Acrophialophora* (from order to genus) and *Westerdykella* (from family to genus). By contrast, *Talaromyces*, *Acrophialophora* and *unclassified_o_Chaetothyriales* were significantly decreased in 0% PEG6000 treated rhizosphere soil; *Chaetomium*, *Acrophialophora* and *Thielavia* were significantly decreased in 10%-PEG6000-treated rhizosphere soil, while at 25% *Sordaria* and *Chloridium* were significantly decreased.

### The GSM inoculation induced greater changes in endophytic fungal communities than the endophytic bacterial communities of *A. lancea*

**Figure 4:**
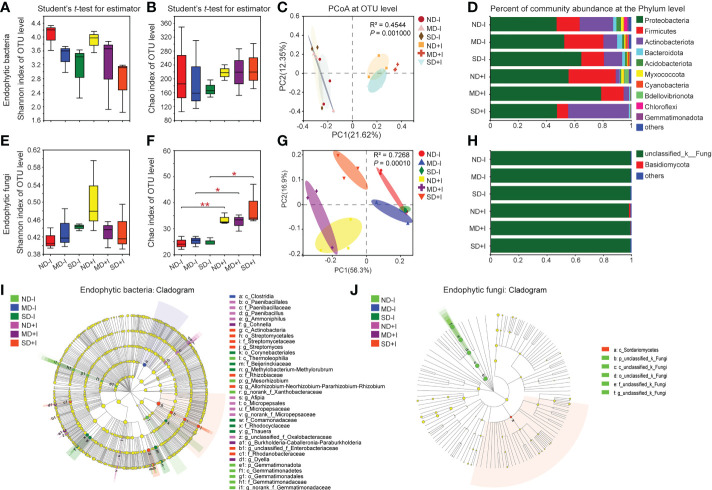
Diversity and community structure of rhizosphere bacteria and fungi of *A. lancea*. Shannon index **(A, E)** shows α diversity, Chao1 index **(B, F)** shows richness, both at the OTU level. C and G: PCoA of endogenetic bacteria **(C)** and Fungi **(G)** in *A. lancea* based on Bray-Curtis and unweighted UniFrac matrix at OTU level, respectively (n = 3). D and H: Bar chart of endogenetic bacteria **(D)** and fungi **(H)** in *A. lancea* endogenetic at phylum level. I and J: Phylogenetic maps of endogenetic bacterial **(I)** and fungal **(J)** lineages of *A. lancea*. Asterisks represent significant difference by Student’s t-test: *P < 0.05; **P < 0.01; ND, no drought stress, 0% PEG6000; MD, mild drought stress, 10% PEG6000; SD, severe drought stress, 25% PEG6000; +I, with Geo-authentic soil microbe inoculation.

We performed 16S rRNA and ITS amplicon sequencing and subsequent comparative analyses on the endophytic bacterial and fungal community structures in the root of the GSM-inoculated and uninoculated *A. lancea* plantlets. The results revealed distinct patterns of alteration in the diversity and the richness of endophytic bacteria and fungi in *A. lancea* roots. The Shannon index showed a decreasing pattern of endophytic bacterial diversity under increasing concentration of PEG6000 (from 0% to 25%), with or without GSM inoculation, although the decrease was not statistically significant (**Figure 4A**). The Chao1 index of the endophytic bacteria in the *A. lancea* root without or post-GSM inoculation remained statistically unchanged under differing PEG6000 concentrations, suggesting a relatively stable endophytic bacterial abundance inside the root (**Figure 4B**). Results of the PCoA on the root endophytic bacteria at the OTU level based on the Bray-Curtis distance algorithm showed that the GSM inoculation was the preliminary factor accounting for differences in the endophytic bacterial communities among sequenced root samples (**Figure 4C**, PC1 = 21.62%).

In the roots of the GSM-inoculated *A. lancea* plantlets grown under normal conditions (0% PEG6000), the phyla Firmicutes (*P* = 0.2958) and Bdellovibrionota (*P* = 0.1027) appeared enriched with higher relative abundance, while the phyla Actinobacteriota (*P* = 0.0362) and Gemmatimonadota (*P* = 0.0339) became depleted and had significantly lower relative abundance. Notably, in the GSM-inoculated *A. lancea* roots, the relative abundance of the phylum Firmicutes generally decreased (10%, *P* = 0.2372; 25%, *P* = 0.0857) while that of the phylum Actinobacteriota (*P* = 0.1027) increased as the concentration of PEG6000 for the treatments increased (**Figure 4D**; **Supplementary Figures 3A–C**), suggesting these phyla might have antagonistic roles in alleviating water deficiency damage. In the endosphere under 0% PEG6000 treatment (ND+I), five groups of bacteria were significantly enriched: *Paenibacillus* (from order to genus), *Ammoniphilus* (from order to genus), *norank_f_Micropepsaceae* (from order to genus), *Afipia* and *unclassified_f_Oxalobacteraceae*. Under 10% PEG6000 treatment (MD+I), three groups of bacteria were significantly enriched: *Cohnella*, *Burkholderia-Caballeronia-Paraburkholderia* (hereinafter referred to as *B-C-P*) and *Dyella*. Finally, under 25% PEG6000 treatment (SD+I), three groups of bacteria were significantly enriched: *Streptomyces* (from class to genus), *A-N-P-R* (from family to genus) and *Rhodanobacteracea*; while endophytic *Paenibacillus* became depleted (*P* = 0.0610) (**Figure 4I**, **Supplementary Figures 3D–F**). Notably, under PEG6000 treatments of multiple concentrations, *A. lancea* preferentially enriched the endophytic genera *B-C-P*, *A-N-P-R* and *Dylla* in its roots but excluded *Comamonas*, *Thauera* and *Sphingomonas* (**Supplementary Figure 3**).

In contrast with the pattern of changes in the diversity of endophytic bacterial communities under PEG6000 treatment (**Figure 4A**), the endophytic fungal communities in the uninoculated *A. lancea* roots showed a weak increasing pattern along with the increasing concentration of PEG6000, although the increase was not statistically significant (**Figure 4E**). Nonetheless, the richness of the endophytic fungi in the GSM-inoculated roots represented by the Chao1 index (**Figure 4F**) was significantly higher compared to that of the uninoculated roots under each treatment. The increased Chao1 index post-GSM-inoculation (**Figure 4F**) demonstrates voluntary enrichment of endophytic fungi by roots when exogenous microbes were provided *via* inoculation. The results of PCoA suggested considerable similarity in the endophytic fungal community structure among the three groups of uninoculated *A. lancea* root samples. GSM inoculation was revealed as an important factor influencing the variance in endophytic fungal community structure (PC1 = 56.3%, as shown in **Figure 4G**).

Comparative analyses of the relative abundance of *A. lancea* root endophytic fungi at the phylum (**Figure 4H**), genus and multi-clade levels (**Figure 4J**) showed that the endophytic fungi in root samples were mainly composed of *unclassified_k_fungi* regardless of whether they were inoculated with GSM or different concentration of PEG6000. The phylum Basidiomycota was specifically enriched in the root of GSM-inoculated *A. lancea* grown under normal conditions (*P* = 0.1339) (**Figure 4H**; **Supplementary Figures 4A–C**). At the OTU level, compared with the uninoculated root, multiple OTUs were significantly and differentially enriched under each treatment. However, due to the lack of phylogenic annotation of fungal OTUs, further interpretation of our data was limited.

### Analyses on the impact of microbial communities on major medicinal compound accumulation in *A. lancea* root

**Figure 5:**
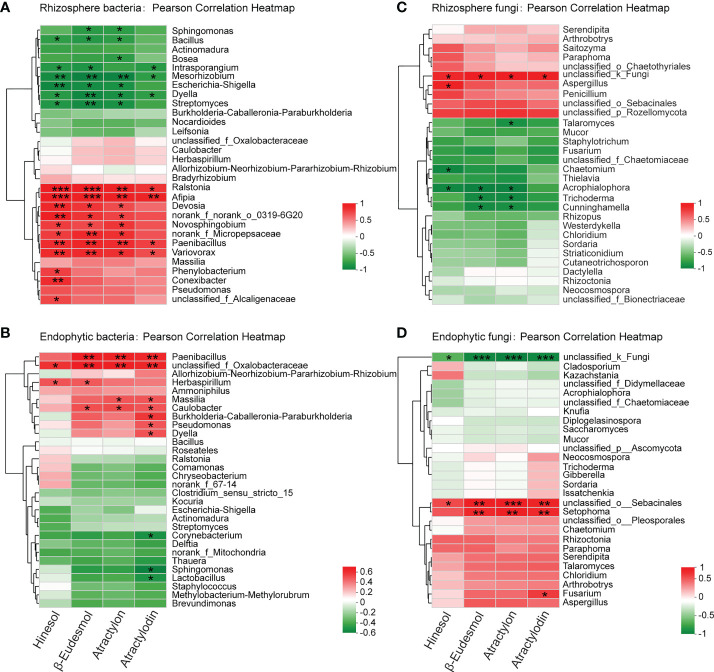
Pearson correlation analysis between the concentration of the four major volatile medicinal compounds in *A lancea* root and the bacteria **(A, B)** and fungi **(C, D)** in *A lancea* rhizosphere and root endosphere at the genus level. * 0.01 < *P* ≤0.05, ** 0.001 < *** *P* ≤ 0.01 by Student’s *t*-test.

To identify the microbial groups that can potentially induce the biosynthesis and accumulation of the medicinal compounds in *A. lancea* root, we performed Pearson correlation analyses on the concentration of the four major medicinal compounds, hinesol, β-eudesmol, atractylon and atractylodin, and the relative abundance of the microbial communities identified in this study (**Figure 5**).

The results showed that at the genus level, the relative abundance of the bacterial genera *Ralstonia*, *Afipia*, *Paenibacillus*, *Variovorax*, *Novosphingobium* and *Devosia* in the rhizosphere of *A. lancea* were positively correlated (*P* < 0.05) with the content of at least three of its four major medicinal compounds, suggesting inducing effects of these bacterial clades on the accumulation of the medicinal compounds (**Figure 5A**). Moreover, inside the root, the endophytic bacterial genus *unclassified_f_Oxalobacteraceae* was positively correlated with all four compounds. The endophytic bacterial genera *Paenibacillus* and *Caulobacter* could potentially induce β-eudesmol, atractylon and atractylodin accumulation. The genera *Massilia*, *B-C-P*, *Pseudomonas* and *Dyella* showed positive correlation with the accumulation of *atractylodin* (**Figure 5B**). Nevertheless, the rhizosphere bacterial genera *Intrasporangium*, *Mesorhizobium*, *Dyella*, *Streptomyces*, *Escherichia-Shigella*, and the endophytic bacterial genera *Corynebacterium*, *Sphingomonas* and *Lactobacillus* had negative correlation with the content of one or more medicinal compounds, indicating possible inhibition of these bacterial groups on the accumulation of medicinal compounds in *A. lancea* root (**Figures 5A, B**).

The fungal genera *Aspergillus* and the *unclassified_k_Fungi* in the rhizosphere were positively correlated with the content of up to four medicinal compounds (**Figure 5C**). Inside the root, the endophytic fungal genera *unclassified_o_Sebacinales*, *Setophoma* and *Fusarium* showed positive correlation with the accumulation of one or more medicinal compounds (**Figure 5C**). The rhizospheric fungal genera *Acrophialophora*, *Trichoderma*, *Chaetomium* and *Talaromyces* were negatively correlated with the accumulation of the four major medicinal compounds of *A. lancea*, the correlation being statistically significant with one to three compounds (**Figure 5C**). One endophytic fungal group identified with the name *unclassified_k_Fungi* had significant negative correlation with all the four major medicinal compounds in *A. lancea* root (**Figure 5D**). A majority of the endophytic fungi were not fully identified and were merely annotated as an unclassified group (**Figures 4H, J**, **5D**), revealing a paucity of current phylogenetic identification information of fungal clades that restricted our findings.

### Verification of endophyte function *via* inoculation

**Figure 6:**
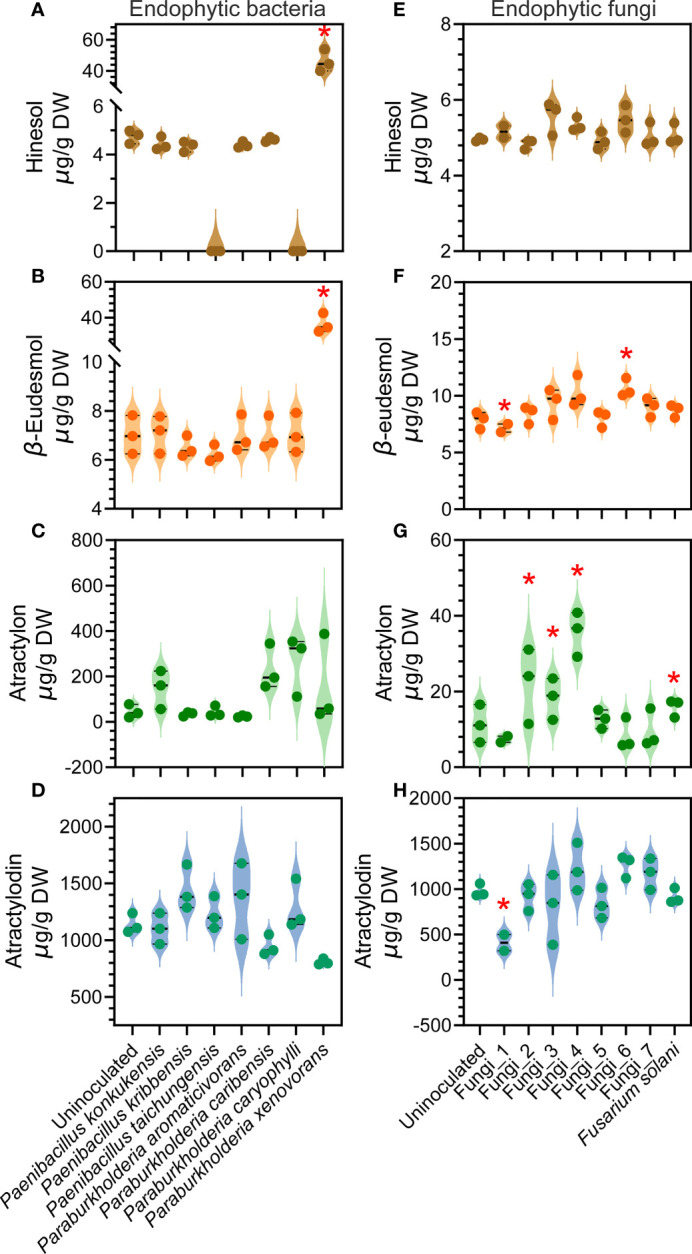
Effects of individual endophytic bacterial or fungal isolates on the concentration of four major medicinal compounds in *A. lancea* root verified *via* inoculation. **(A–D)** results of endophytic bacteria inoculation; **(E–H)** results of endophytic fungi inoculation. Asterisks represent significant difference compared with the uninoculated plantlets by Student’s t-test: *P < 0.05.

Multiple studies have now reported the inducing effects of specific endophyte isolates microorganisms on the accumulation of the major medicinal compounds in *A. lancea* (Ren and Dai, 2012; Wang et al., 2015; Zhou et al., 2016). Previously only a handful of studies had reported rhizosphere microbial isolates microorganisms with such functions. Hence, in this study, we isolated rhizome endophytes from the wild *A. lancea* collected at the identical site where the soil used for the GSM inoculum was collected in order to verify their function. It was found that the endophytic bacterial genera *Paenibacillus* is positively correlated with the accumulation of the major medicinal compounds in the root (**Figure 5B**). We performed inoculation assays using a total of three strains of *Paenibacillus* to separately verify whether they could induce the production of hinesol, β-eudesmol, atractylon or atractylodin. The strains used were *P. konkukensis*, *P. kribbensis*, and *P. taichungensis*. The results showed mild inducing effects of the *P. konkukensis* strain on the production of β-eudesmol and atractylon, and mild inducing effects of the *P. kribbensis* strain and the *P. taichungensis* strain on atractylodin accumulation (**Figures 6A–D**).

The root endophytic *B-C-P* spp. were found to be positively correlated with the accumulation of β-eudesmol, atractylon and atractylodin, with only the correlation with atractylodin accumulation statistically significant (**Figure 5B**). Inoculation assays using a total of four endophytic *Paraburkholderia*, namely *P. aromaticivorans*, *P. caribensis*, *P. caryophylli* and *P. xenovorans*, allowed us to individually verify the inducing effects on medicinal compound accumulation. The results revealed a significant inducing effect of the *P. xenovorans* strain on hinesol and β-eudesmol accumulation, as well as differential inducing effects of the other three endophytic *Paraburkholderia* strains on β-eudesmol, atractylon or atractylodin accumulation (**Figure 6A–D**). These results demonstrated that the effects of individual endophytic bacterial strains are distinct, indicating specific function of different bacterial strains within a genus. Promoting effects were revealed for certain strains, suggesting consistency to an extent with the results of our correlation analyses (**Figure 5B**).

From the endophytic fungal genera that we identified as potential inducers of medicinal compound accumulation (**Figure 5D**). We managed to isolate 7 endophytic fungi without clear classification and one strain corresponding to a fungal genus positively correlated to medicinal compound accumulation, namely *Fusarium solani* strain (**Figure 5D**). We named the unclassified endophytic fungal strains eFungus_1 to eFungus_7. We performed inoculation assays using these endophytic fungal strains (**Figures 6E–H**). eFungus_1 significantly inhibited the accumulation of β-eudesmol and atractylodin. eFungus_2, eFungus_3, eFungus_4 and *Fusarium solani* promoted accumulation of atractylon. eFungus_6 had a significant promoting effect on β-eudesmol.

## Discussion

### The GSM inoculation promoted the growth and drought-resistant phenotype of *A. lancea*

The number of adventitious roots, root length, aerial biomass, root biomass and root dry mass all increased after GSM inoculation under each treatment (**Figures 2A–E**), demonstrating the growth promoting effects of the GSM. Such growth promoting effects likely resulted from the synergistic action of the GSM, which may contain multiple microbe communities or strains beneficial for *A. lancea* growth.

Moreover, the phenotype of the *A. lancea* plantlets was altered by GSM inoculation and PEG6000 treatments. Biomass measurements of uninoculated plantlets showed inhibition of *A. lancea* growth under 10% PEG6000 treatment and more severe impairment of growth under 25% treatment. However, the root dry mass accumulation appeared slightly improved under 25% PEG6000 treatment compared to either 0% or 10% (*P* = 0.045 and 0.174, respectively, **Figure 2A**); meanwhile, the root:shoot ratio was significantly increased (**Figure 2B**). A relatively large allocation of biomass into the root is a characteristic trait associated with the perennial growth form; in many plant species, this trait is also associated with drought tolerance (Chapin et al., 1993; Comas et al., 2013). As a perennial plant, the uninoculated *A. lancea* plantlets developed the phenotype with significantly increased root:shoot biomass ratio after exposure to 25% PEG6000 treatment conditions (**Figure 2C**), suggesting this trait was closely associated with their drought tolerance. These results suggest GSM inoculation promoted a drought-resistant phenotype in *A. lancea*.

The water content in the root is also an important indicator of plant fitness and drought tolerance. Drought-resistant cultivars can maintain a relatively high water content under water-deficient treatments (Maghsoudi et al., 2016). In this study, the GSM inoculation significantly improved the DW-based water content of *A. lancea* root under 0% and 10% PEG6000 treatment. Under the extreme water deficiency inflicted by 25% PEG6000, the GSM inoculated pliantly maintained higher root water content compared to the uninoculated *A. lancea*. The relatively stable water content data under PEG6000 treatments post-GSM-inoculation showed improved root capacity to hold moisture (**Figure 2E**). Taken together, the improved growth and drought-resistance of GSM inoculated *A. lancea* may translate to increased agricultural yield and better adaptation to climate change.

### The GSM improved medicinal compound accumulation in *A. lancea* root but was susceptible to PEG6000 stress inhibition

Under normal growth conditions, the GSM inoculation generally improved the accumulation of the four major volatile medicinal compounds, namely atractylon, β-eudesmol, atractylon and atractylodin (**Figures 2F–I**). Unlike in our previous study looking at the effects of GSM inoculation on *A. lancea* under heat stress we did not observe increased hinesol accumulation (**Figure 2F**) (Wang et al., 2022). Interestingly, without GSM inoculation water deficiency stress did not alone induce increases in any of the four compounds, in the way heat stress did for β-eudesmol (Wang et al., 2022). This suggests that the metabolism of each compound is differentially impacted by distinct environmental factors. The promoting effects of the GSM decreased as the concentration of PEG6000 treatment increased, suggesting that the GSM is itself susceptible to water deficiency stress (**Figures 2A, B**, **3A, J**, **4A**, **3J**).

These results could also reflect a resource allocation and investment pattern in *A. lancea* whereby survival is prioritized over secondary metabolite accumulation under environmental stress. The growth–differentiation balance hypothesis suggests plantlets increase or decrease resource allocation to secondary metabolism in response to environmental conditions. The key premise of this hypothesis is that trade-offs constantly occur between the primary and secondary metabolism of plantlets (Herms and Mattson, 1992; Hale et al., 2005). When the environmental stress is severe enough to depress the carbon assimilation of plantlets, plant growth slows down due to limitation in resources. In such cases, the secondary metabolism falls due to low energy and substrates availability to supply secondary metabolite biosynthesis (Hale et al., 2005). In this study, the 10% and 25% PEG6000 may have suppressed the carbon assimilation of *A. lancea*, consistent with their significantly inhibited growth.

The suppression of the growth and drought resistance promoting effects of GSM by water deficiency stress suggests that, while microbe mixtures have great potential for application in *A. lancea* farming, water regimens will need to be carefully managed for their full benefit to be realized.

### Fungal communities were less impacted by PEG6000 treatment than bacterial communities

In this study, we revealed that the bacterial communities in the rhizosphere of *A. lancea* or the bottled soil were strongly impacted by PEG6000 treatment (**Figure 3C**). The diversity and richness of the rhizosphere bacteria were negatively correlated with PEG6000 concentration (**Figures 3A, B**). The opposite was true for fungi (**Figures 3E–G**). These results indicate that a considerable proportion of the bacteria in the GSM are drought-susceptible, the fungi are relatively drought-resistant. It has previously been reported that different components of soil microbial communities respond differently to drought. A previous study on sugarcane-root-associated bacteria reported that drought stress significantly reduced the overall bacterial diversity while only increasing the abundance of drought-resistant bacteria in the sugarcane rhizosphere (Liu et al., 2021). Soil bacterial networks are known to be less stable than fungal networks under drought stress. Soil fungi are generally more resistant than bacteria to drought (Bapiri et al., 2010; Barnard et al., 2013; De Vries and Shade, 2013; De Vries et al., 2018). Our results were consistent with these previous findings. Rhizosphere fungi may provide relatively stronger protection *A. lancea* plantlets under drought stress than the rhizosphere bacteria.

The metabolism of plants can fluctuate considerably when subjected to abiotic stresses (Buffagni et al., 2020; Ahmed et al., 2021), which might in turn modify the endophytic microbe communities (Bhattacharyya et al., 2021). This means that plants can take the initiative selecting the microbial communities that they harbor within their roots by varying the metabolites contained in their roots. In *A. lancea* root, the endophytic bacterial communities remained relatively stable under different PEG6000 concentrations (**Figures 4A, C**). The richness of the endophytic bacteria also increased, but this was not statistically significant (**Figure 4B**). However, the richness of the endophytic fungi was significantly promoted after the GSM inoculation (**Figure 4F**). These results suggest that GSM inoculation was an important source from which *A. lancea* acquired and enriched microbes in its root. *A. lancea* selectively enhanced the endophytic fungi richness in its root, indicating they have a crucial role in promoting drought-resistant traits. Collectively, the community structure formation of the fungi from the GSM inoculum turned out to be less affected by PEG6000 concentration but more responsive to the physiological status of the *A. lancea* plantlets compared to the bacteria.

### Drought stress can impact *A. lancea* growth and medicinal compound accumulation *via* modifying the abundance of specific bacterial clades

Our comparative analyses of the relative abundance of the microbe communities at the phylum level and the cladograms showing differentially enriched microbe groups at multi-clade levels have revealed a number of microbe clades that may enhance drought resistance in *A. lancea* (**Figures 3**–**5**; **Supplementary Figures 1–4**). Among them, many bacterial clades have been associated with plant drought resistance in previous studies.

Collectively, our results show that at the genus level, *A. lancea* specifically enriched *Massilia* and *A-N-P-R* in its rhizosphere. However, *Massilia* could resist 10% PEG6000 drought but was severely reduced under 25% PEG6000 treatment. By contrast, *A. lancea* showed the tendency to specifically reduce the relative abundance of *B-C-P* from in its rhizosphere. Nevertheless, *B-C-P* appeared highly drought-resistant and had high relative abundance in the GSM, hence, it maintained high abundance in rhizosphere at all PEG6000 concentrations. Moreover, since *B-C-P* was highly abundant in all soil samples, the detected decrease in relative abundance of *B-C-P* can be attributed to the plant enriching other genera, rather than to *B-C-P* being selectively excluded. This is supported by the unchanged ranking of *B-C-P* relative abundance under all treatments. Notably, the genera *Pseudomonas*, *Paenibacillus*, *Conexibacter* and *Ralstonia* also appeared drought-sensitive, with their relative abundance decreasing as the concentration of PEG6000 increased. *A. lancea* specifically enriched different bacterial communities under 10% and 25% PEG treatments, which might have been strategies to cope with water deficiency stress of varying intensity in collaboration with soil microbes. *Bacillus*, *Dylla* and *Actinomadura* became highly enriched in *A. lancea* rhizosphere specifically under 25% PEG6000 treatment, suggesting these genera are crucial bacterial clades that protect *A. lancea* under severe drought stress. The role of *Bacillus* spp. in inducing stress resistance in crops is well-documented (Radhakrishnan et al., 2017). *Actinomadura* of the phylum Actinobacteria have been reported as a specific drought-enriched bacterial genus (Xu et al., 2018). The phylum Actinobacteria is often specifically enriched in relatively dry environments or under drought conditions (Xu et al., 2018; Kumar et al., 2022). *Actinobacteria* was reported to be crucial for a drought-sensitive sugarcane cultivar GT39 to cope with drought stress, while a relatively drought-resistant sugarcane cultivar ZZ9 relied mainly on Bacilli to adapt to drought (Liu et al., 2021). A study looking at rice root-associated microbiota found that changes in bacterial communities in response to drought were taxonomically consistent across soils and different rice cultivars and were primarily driven by the enrichment of the genera *Actinobacteria* and *Chloroflexi* and, depletion of several *Deltaproteobacteria* species (Santos-Medellín et al., 2017). Similarly, we found *Chloroflexi* depleted in the rhizosphere of *A. lancea* in response to drought in a manner that may be plant-specific.

Our findings reveal that the structure of the endophytic bacterial communities remained relatively stable compared to the rhizosphere (Figs. 3A-3C, 4A-4C). Nonetheless, *A. lancea* selectively and flexibly favored the enrichment of different bacterial clades in its rhizosphere or root endosphere under drought stress of varied severities. Comparative analyses on the endophytic bacterial communities suggest that the genera *Actinomadura* and *A-N-P-R* were specifically enriched thus important for protecting *A. lancea* from drought conditions as severe as 25% PEG6000 treatment, while *Massilia* and *B-C-P* may be important under less severe drought conditions represented by the 10% PEG6000 treatment. *A. lancea* tended to enrich *B-C-P* in its root endophytes while increasing *B-C-P* abundance in its rhizosphere. The bacterial genus *A-N-P-R* also appeared favored by *A. lancea* in both the rhizosphere and root endosphere. In contrast, *A. lancea* appeared to prefer *Sphingomonas* specifically in its rhizosphere rather than its root endosphere. Notably, *A. lancea* would prefer specific enrichment of *Paenibacillus* in its root endosphere. *Paenibacillus* spp. are widely reported as plant-growth-promoting bacteria and have antagonistic activity against phytopathogens (Grady et al., 2016; Rybakova et al., 2016). However, due to the drought-sensitivity of *Paenibacillus*, as well as possibly due to *A. lancea*’s need of protection from particular drought-associated bacterial genera including endophytic *B-C-P*, *A-N-P-R*, *Dylla*, and rhizospheric *Bacili* and *Dylla*, *Paenibacillus* abundancy becomes depleted under drought stress. To summarize, our results showed the bacterial community structure was sensitive to drought; hence, it can be postulated that drought directly affected the bacterial communities, which in turn affected the growth and medicinal compound accumulation of *A. lancea*.

### Novel roles of specific fungal clades in protecting *A. lancea* from drought stress were revealed

Our finds on the fungal communities showed that the genera *Chaetomium* and *Acrophialophora* were preferentially enriched by *A. lancea* in its rhizosphere under PEG6000 treatments although they were not identified as drought-resistant fungi (**Supplementary Figure 8**), suggesting their important roles in protecting *A. lancea* from drought stress. Notably, the relative abundance of the genera *Acrophialophora*, *Trichoderma* and *Thielava* increased as the concentration of the PEG6000 treatment increased, suggesting they are the key fungal groups that protected *A. lancea* under drought stress (**Supplementary Figure 8**). Fungi of the genus *Trichoderma* are widely known for promoting plant growth and enhancing the stress resistance of plantlets (Hermosa et al., 2012; Topolovec-Pintarić, 2019). A few *Acrophialophora* spp. have also been reported to have antagonistic effects against plant pathogens and enhance the tolerance of host plantlets, although most of these beneficial effects were reported for endophytic *Acrophialophora* strains (Zhou et al., 2015; Daroodi et al., 2021a; Daroodi et al., 2021b). Fungi of the *Thielava* genus have seldom been subject to research and to the best of our knowledge, we have reported its association with drought resistance in *A. lancea* for the first time in the present study.

In the endosphere of the *A. lancea* root, the endophytic fungal communities remained relatively unchanged between different samples, as did the endophytic bacterial communities. Notably, endophytic *Fusarium* appeared enriched in the GSM inoculated *A. lancea* root under 25% PEG6000 treatment (**Supplementary Figure 4**). A majority of the fungi of the genus *Fusarium* are soil borne plant pathogens that cause root rot and wilting and severely impair plant and crop health (Kidd et al., 2011; Xiong et al., 2017). However, some *Fusarium* species have also been reported to promote plant growth *via* particular signaling chemicals or phytohormone derivates they produce (Bitas et al., 2015; Bilal et al., 2018). For *A. lancea*, free-living *Fusarium* spp. are well known as pathogens causing devastating root rot disease (Ren et al., 2017; Li et al., 2018). Our results reveal that *Fusarium* also becomes enriched in *A. lancea* rhizosphere under drought conditions (**Supplementary Figure 8**), suggesting it may have a role in improving the drought resistance of *A. lancea*. The significance and function of *Fusarium* spp. on the medicinal plant *A. lancea* is worthy of further study.

### Particular microbe isolates within a certain genus might have different functions

The results of this study revealed the genus *Paenibacillus*, both free-living in the rhizosphere and endophytic in the root, as a key microbe clade for *A. lancea* fitness under stress and its medicinal compound accumulation. Meanwhile, the bacterial genus *B-C-P*, which was highly abundant as free-living bacteria both in the soil and in the rhizosphere of *A. lancea*, was shown to play a more important role in root endosphere than in the soil. Hence, we selected the endophytic strains of these two genera from all the bacterial endophytes we isolated and performed inoculation assays to investigate their function on the accumulation of each major medicinal compound in *A. lancea* root. Additionally, we verified the function of the one endophytic *Fusarium* strain we isolated. The results showed that only certain strains could significantly induce the accumulation of particular medicinal compound(s) (**Figure 6**). Indeed, the microbial groups that we identified to potentially promote drought resistance and medicinal compound accumulation were acquired based on the collective outcome of massive microbe strains, not the effects of individual strains. The ultimate purpose of our study is to contribute to the future isolation and deployment of microbial agents that promote *A. lancea* growth, stress resistance and medicinal value. Our results suggest it is important to verify the function of specific microbe strains individually. Development and application of combinations of multiple microbial agents would be optimal to achieve improved outcomes in *A. lancea* farming.

## Conclusion

In this study, we reveal that the inoculation with GSM was beneficial for *A. lancea* growth, medicinal compound accumulation and adaptation to drought. *A. lancea* selectively enriches specific clades of bacteria and fungi under drought stress of varied severities, partially due to the drought sensitivity of some microbes. Drought can be a more powerful driving force that shapes the root-associated microbe communities than the selectivity of A. lancea, especially for the rhizospheric bacteria. The community structure of rhizospheric fungi was more stable than bacteria, suggesting fungi were more resistant to drought; also, higher abundance of endophytic fungi than endophytic bacteria was found in *A. lancea* when subjected to drought stress. Hence, we conclude that fungi have a stronger role in protecting *A. lancea* from drought stress. Overall, the bacterial genera *Bacillus*, *Dylla*, *Actinomadura*, *B-C-P* and *A-N-P-R*, and the fungal genera *Chaetomium*, *Acrophialophora*, *Trichoderma*, *Thielava* and *Fusarium* were identified as the crucial root-associated microbial clades that protected *A. lancea* from drought stress. We verified that the effects of different endophytic *Paenibacillus*, *Paraburkholderia* and *Fusarium* strains of each genus were differential. Hence, besides identifying the crucial microbial clades that benefit *A. lancea* under stress, it is equally important to verify the function of each microbe isolate *via* inoculation for future application of microbial agents on *A. lancea* cultivation.

## Data availability statement

The original contributions presented in the study are publicly available. This data can be found here: NCBI, PRJNA806199.

## Author contributions

All authors contributed to the conception and design of this study. Material preparation, data measurements and analyses were performed by Wang HY, Wang YF, Kang CZ, and Xiang ZX. The first draft of the manuscript was written by Wang HY and Wang YF. The latest version of the manuscript was written by Wang YF. Wang HY and Wang YF made the figures. All authors gave valuable suggestions on each version of the manuscript; all authors read and approved the final manuscript.

## Funding

This work was financially supported by National Natural Science Foundation of China (No: 81891014); Scientific and technological innovation project of China Academy of Chinese Medical Sciences (CI2021A03903, CI2021A03905, CI2021B013); Innovation Team and Talents Cultivation Program of National Administration of Traditional Chinese Medicine. (No: ZYYCXTD-D-202005); China Agriculture Research System of MOF and MARA (CARS-21); Key Project at Central Government level: the ability establishment of sustainable use for valuable Chinese medicine resources (2060302).

## Conflict of interest

The authors declare that the research was conducted in the absence of any commercial or financial relationships that could be construed as a potential conflict of interest.

## Publisher’s note

All claims expressed in this article are solely those of the authors and do not necessarily represent those of their affiliated organizations, or those of the publisher, the editors and the reviewers. Any product that may be evaluated in this article, or claim that may be made by its manufacturer, is not guaranteed or endorsed by the publisher.
