# Supplementary material for: Drought stress modifies the community structure of root-associated microbes that improve Atractylodes lancea growth and medicinal compound accumulation
Source: Front Plant Sci. 2022 Dec 2;13:1032480. doi: 10.3389/fpls.2022.1032480 (PMC9756954; doi:10.3389/fpls.2022.1032480)
Supplement: Supplementary file 1 [file DataSheet_1.docx]

**Supplementary Material**

**
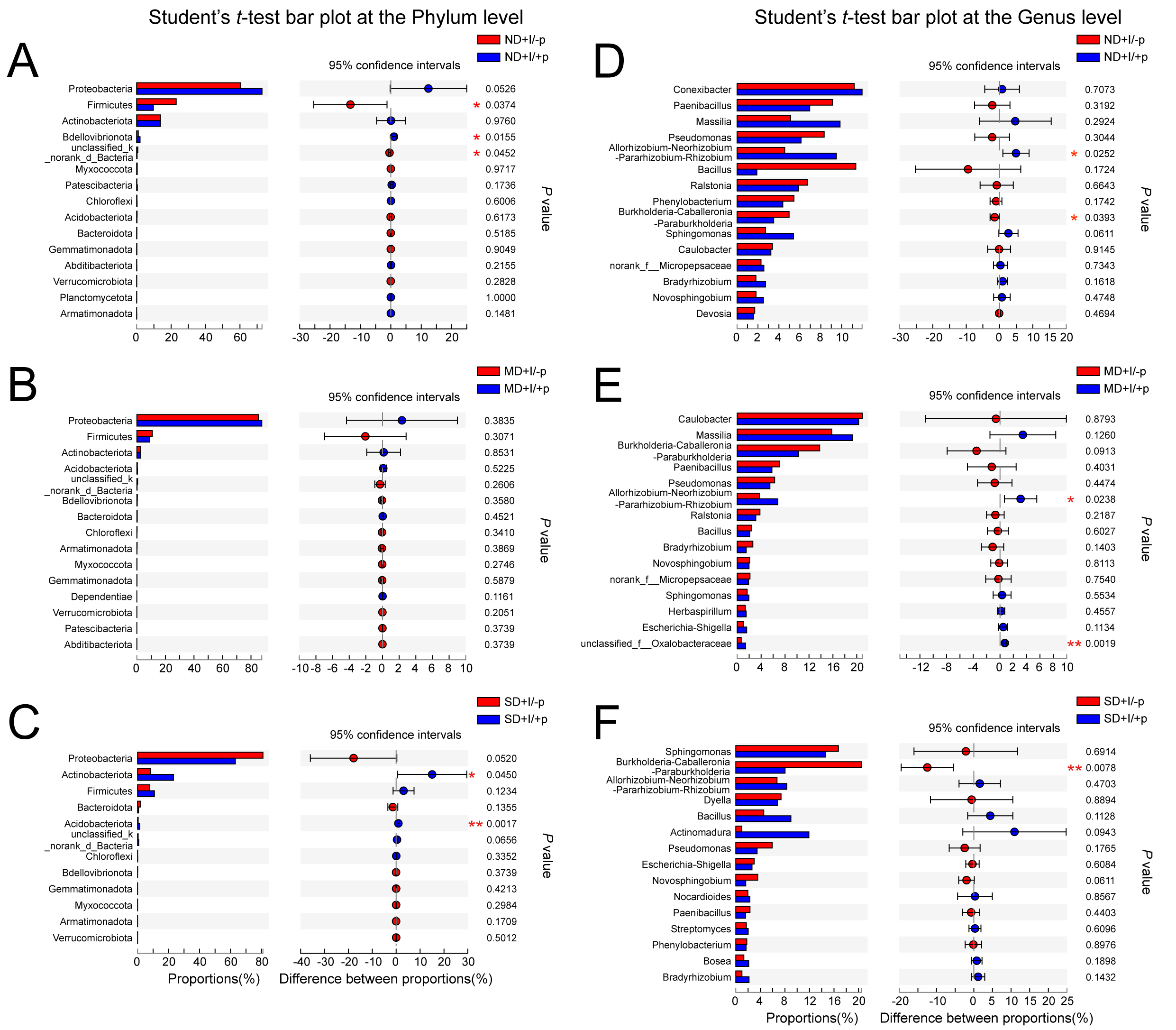
**

**Supplementary Figure 1.** Comparison of the relative abundance of the top 15 most abundant bacterial phyla (A-C) or genera (D-F) of the bottled soil and *A. lancea* rhizosphere soil undergone different PEG6000 treatments (n = 3). Asterisks represent significant difference by Student’s *t*-test: * *P* < 0.05; ** *P* < 0.01; *** *P* < 0.001. ND: no drought stress, 0% PEG6000; MD: mild drought stress, 10% PEG6000; SD: severe drought stress, 25% PEG6000; +I, with Geo-authentic soil microbe inoculation; +p, with *A. lancea* plantlet.


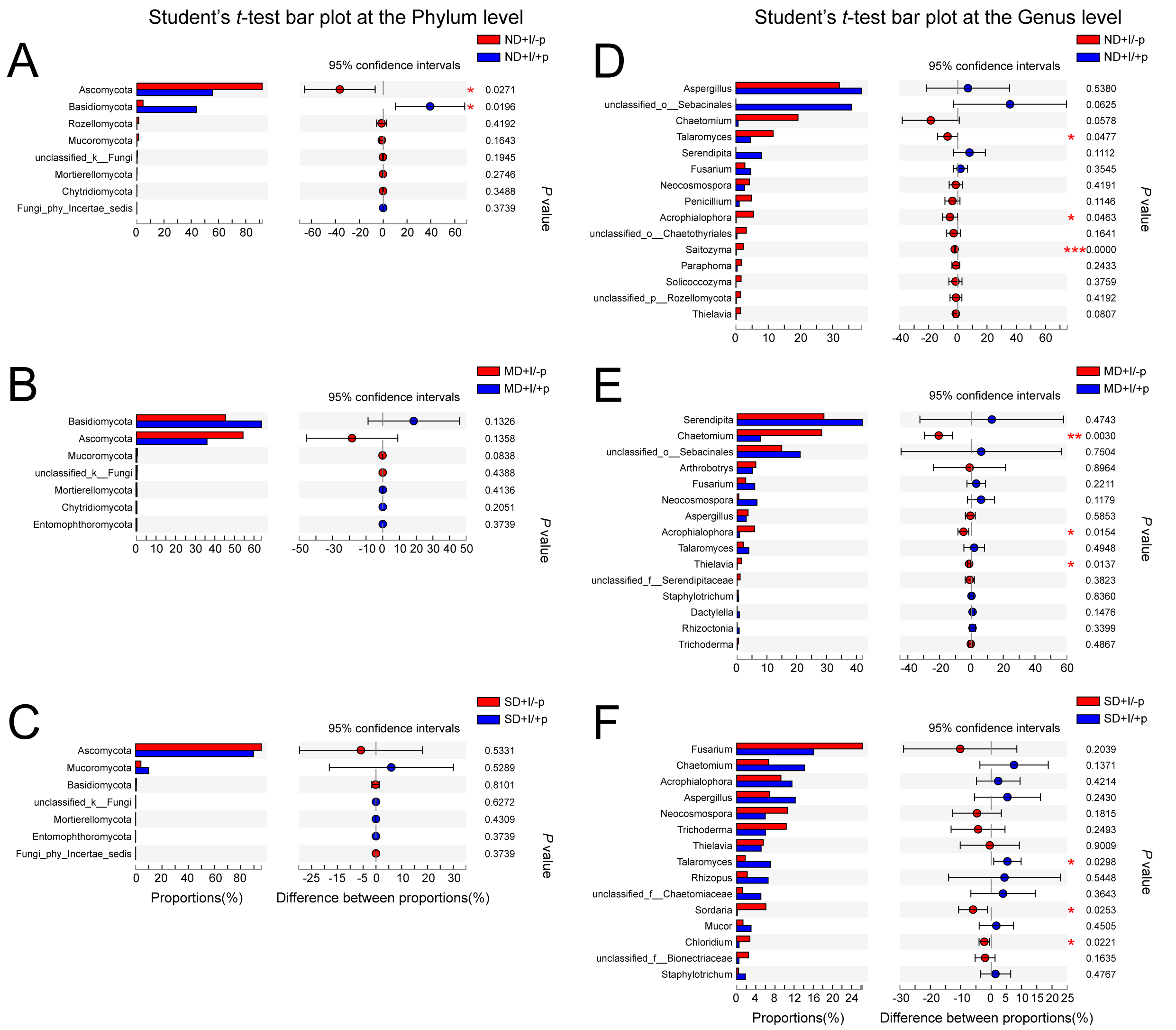


**Supplementary Figure 2.** Comparison of the relative abundance of the top 15 most abundant fungal phyla (A-C: not reaching 15 phyla in total) or genera (D-F) of the bottled soil and *A. lancea* rhizosphere soil undergone different PEG6000 treatments (n = 3). Asterisks represent significant difference by Student’s *t*-test: * *P* < 0.05; ** *P* < 0.01; *** *P* < 0.001. ND: no drought stress, 0% PEG6000; MD: mild drought stress, 10% PEG6000; SD: severe drought stress, 25% PEG6000; +I, with Geo-authentic soil microbe inoculation; +p, with *A. lancea* plantlet.


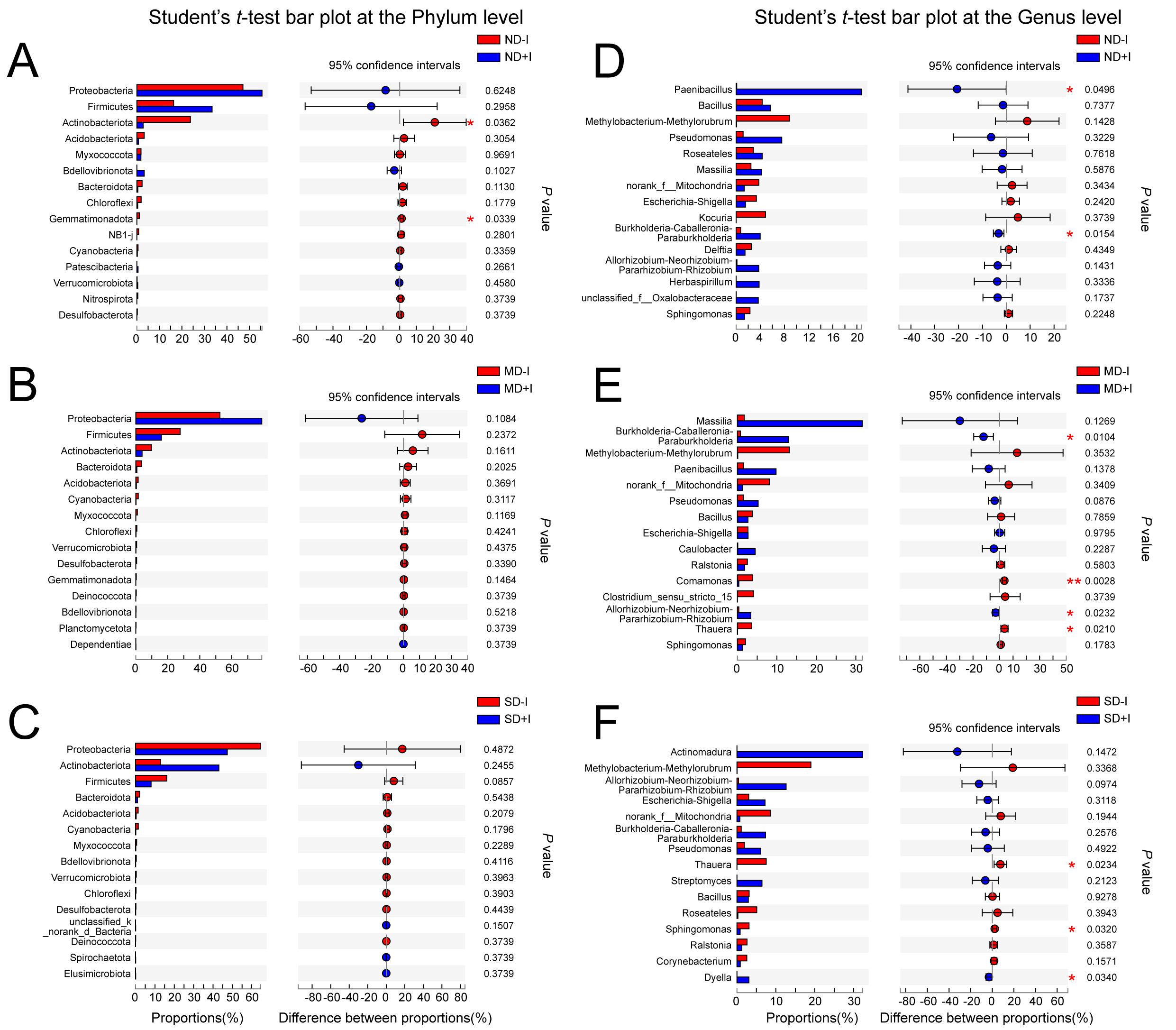


**Supplementary Figure 3.** Comparison of the relative abundance of the top 15 most abundant bacterial phyla (A-C) or genera (D-F) of *A. lancea* root endophytic bacteria with or without GSM inoculation undergone different PEG6000 treatments (n = 3). Asterisks represent significant difference by Student’s *t*-test: * *P* < 0.05; ** *P* < 0.01; *** *P* < 0.001. ND: no drought stress, 0% PEG6000; MD: mild drought stress, 10% PEG6000; SD: severe drought stress, 25% PEG6000; +I, with Geo-authentic soil microbe inoculation.


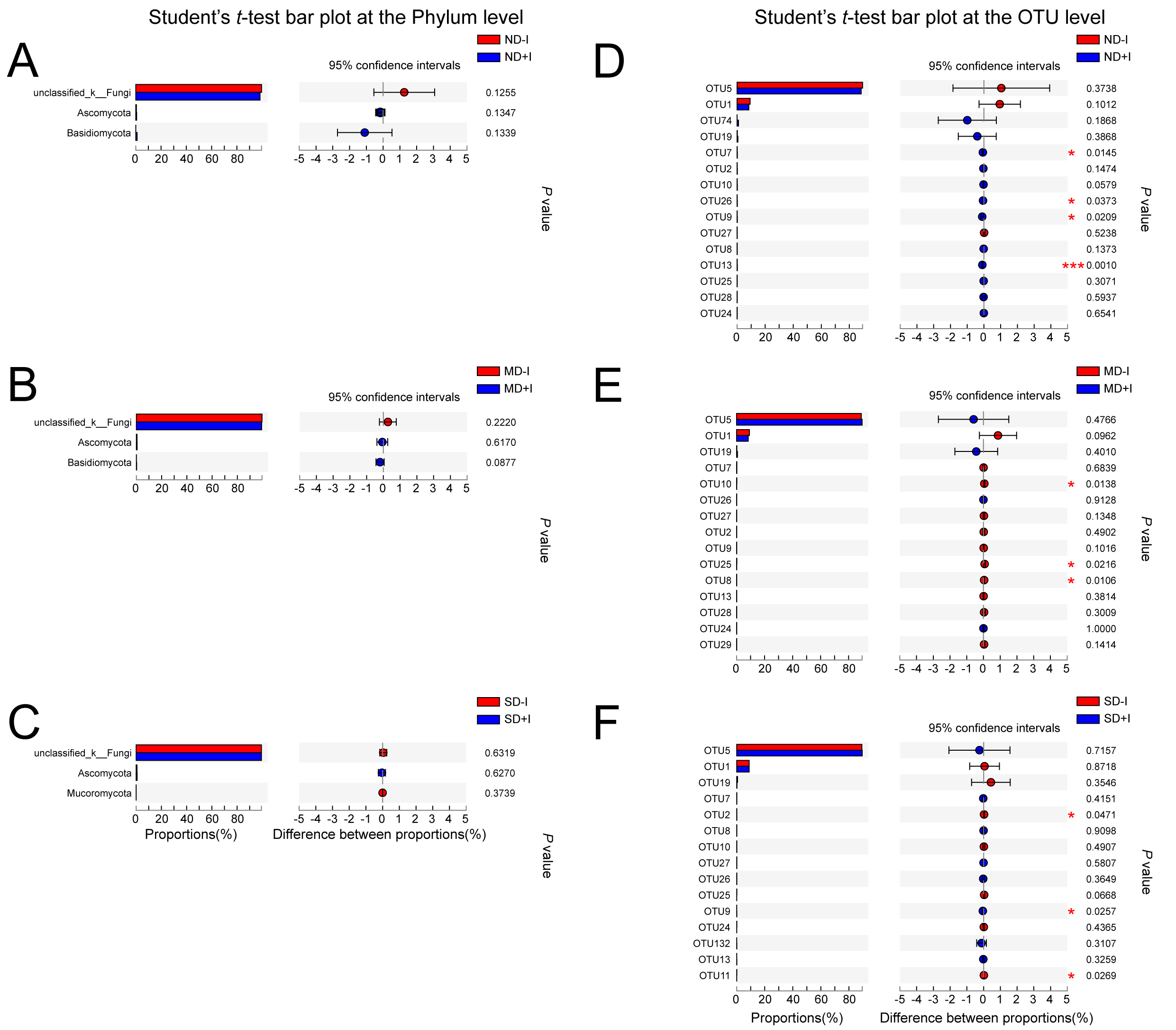


**Supplementary Figure 4.** Comparison of the relative abundance of the top 15 most abundant fungal phyla (A-C) or OTU (D-F) of *A. lancea* root endophytic fungi with or without GSM inoculation undergone different PEG6000 treatments (n = 3). Asterisks represent significant difference by Student’s *t*-test: * *P* < 0.05; ** *P* < 0.01; *** *P* < 0.001. ND: no drought stress, 0% PEG6000; MD: mild drought stress, 10% PEG6000; SD: severe drought stress, 25% PEG6000; +I, with Geo-authentic soil microbe inoculation.


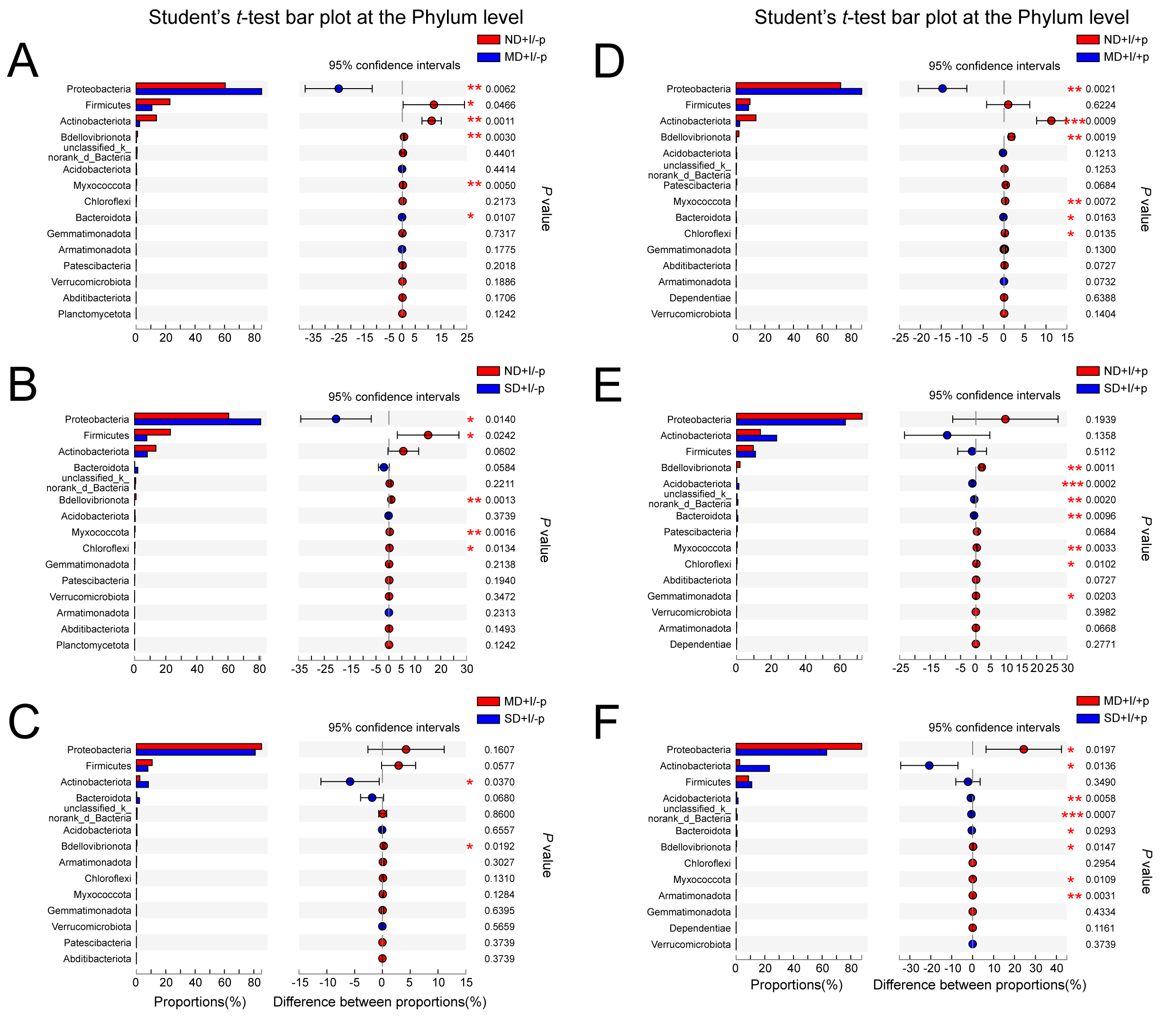


**Supplementary Figure 5.** Comparison of the relative abundance of the top 15 most abundant bacterial phyla of the bottled soil (A-C) and *A. lancea* rhizosphere soil (D-F) undergone different PEG6000 treatments (n = 3). Asterisks represent significant difference by Student’s *t*-test: * *P* < 0.05; ** *P* < 0.01; *** *P* < 0.001. ND: no drought stress, 0% PEG6000; MD: mild drought stress, 10% PEG6000; SD: severe drought stress, 25% PEG6000; +I, with Geo-authentic soil microbe inoculation; +p, with *A. lancea* plantlet.


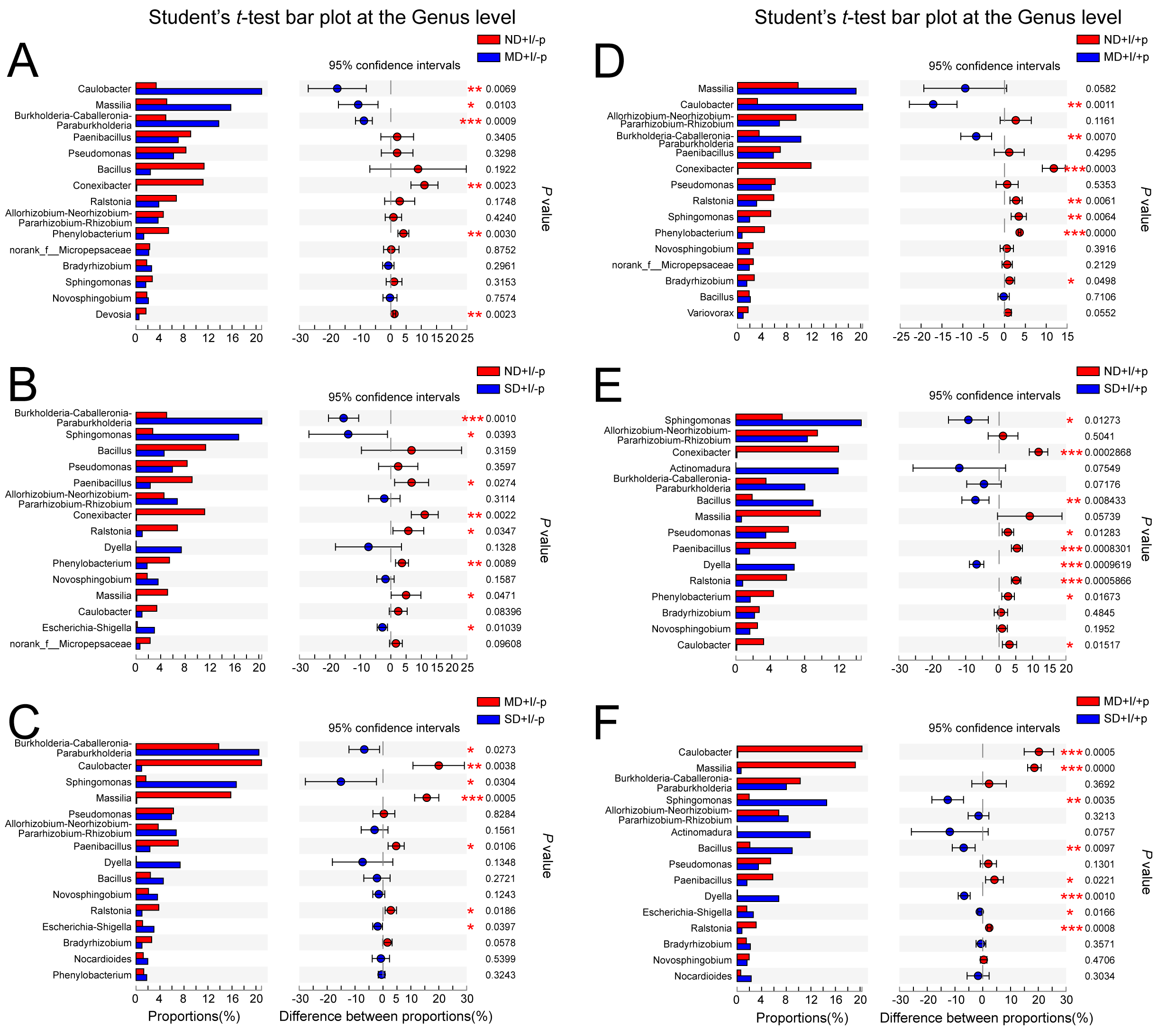


**Supplementary Figure 6.** Comparison of the relative abundance of the top 15 most abundant bacterial genera of the bottled soil (A-C) and *A. lancea* rhizosphere soil (D-F) undergone different PEG6000 treatments (n = 3). Asterisks represent significant difference by Student’s *t*-test: * *P* < 0.05; ** *P* < 0.01; *** *P* < 0.001. ND: no drought stress, 0% PEG6000; MD: mild drought stress, 10% PEG6000; SD: severe drought stress, 25% PEG6000; +I, with Geo-authentic soil microbe inoculation; +p, with *A. lancea* plantlet.


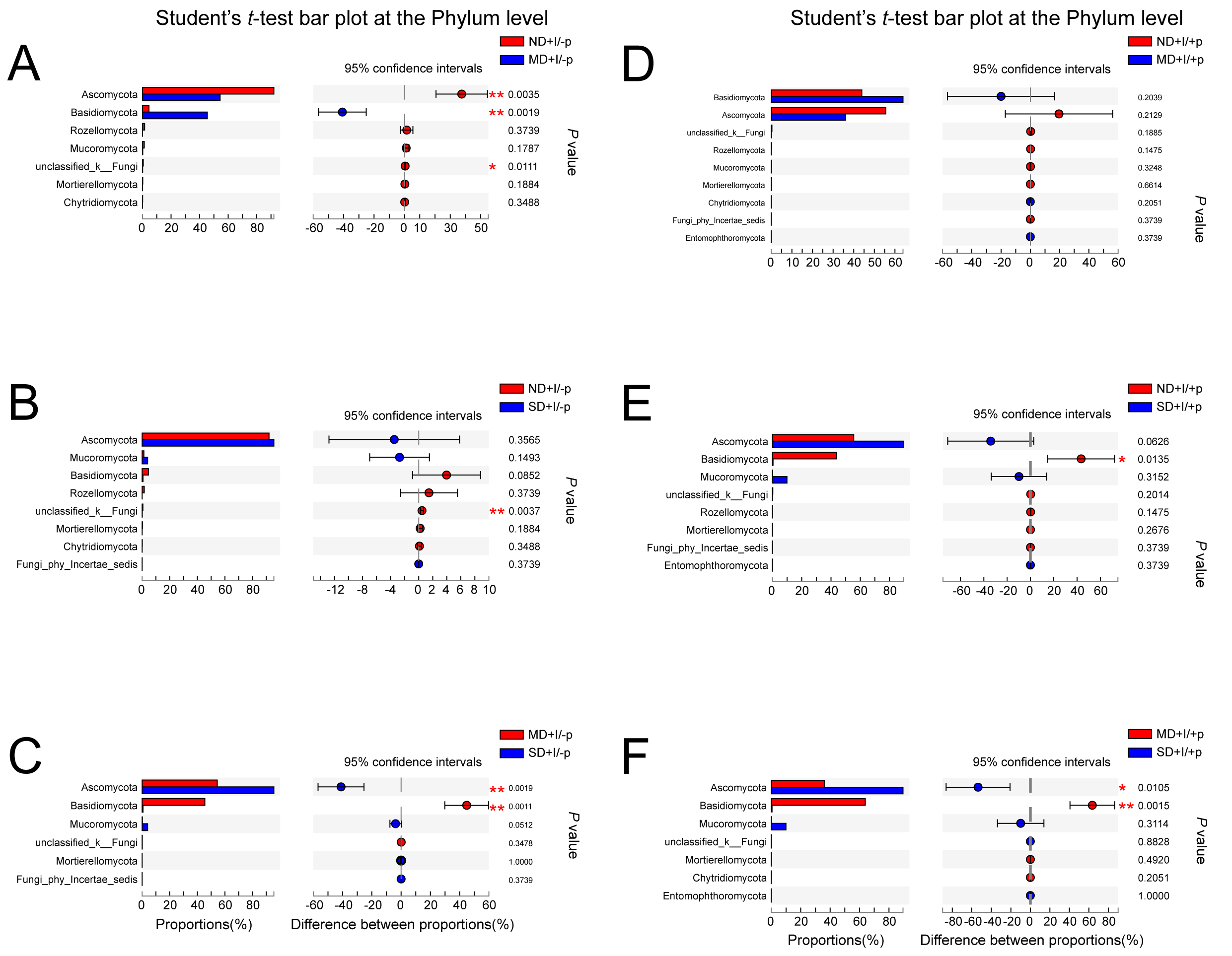


**Supplementary Figure 7.** Comparison of the relative abundance of the top 15 most abundant fungal phyla of the bottled soil (A-C) and *A. lancea* rhizosphere soil (D-F) undergone different PEG6000 treatments (n = 3). Asterisks represent significant difference by Student’s *t*-test: * *P* < 0.05; ** *P* < 0.01; *** *P* < 0.001. ND: no drought stress, 0% PEG6000; MD: mild drought stress, 10% PEG6000; SD: severe drought stress, 25% PEG6000; +I, with Geo-authentic soil microbe inoculation; +p, with *A. lancea* plantlet.


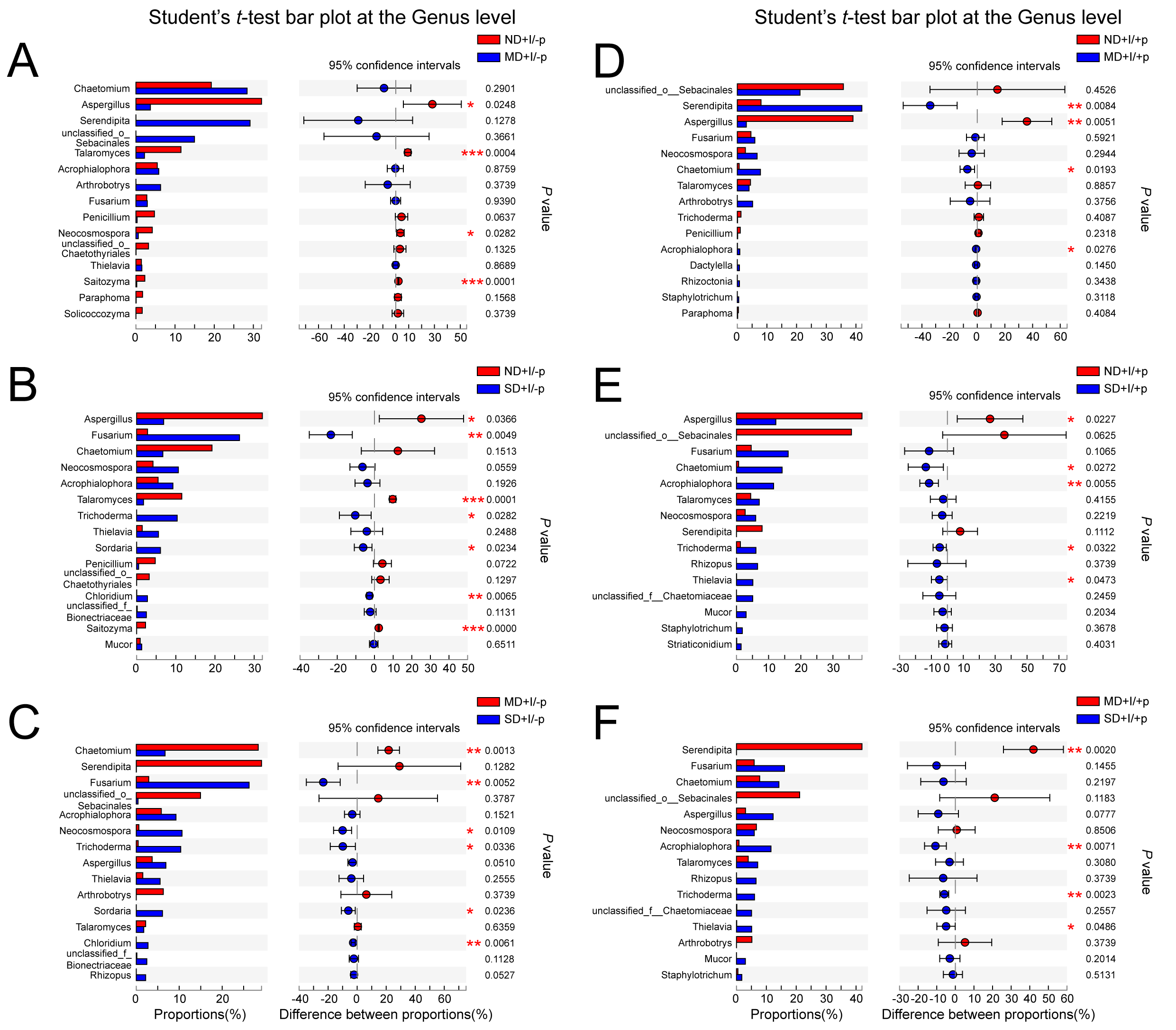


**Supplementary Figure 8.** Comparison of the relative abundance of the top 15 most abundant fungal genera of the bottled soil (A-C) and *A. lancea* rhizosphere soil (D-F) undergone different PEG6000 treatments (n = 3). Asterisks represent significant difference by Student’s *t*-test: * *P* < 0.05; ** *P* < 0.01; *** *P* < 0.001. ND: no drought stress, 0% PEG6000; MD: mild drought stress, 10% PEG6000; SD: severe drought stress, 25% PEG6000; +I, with Geo-authentic soil microbe inoculation; +p, with *A. lancea* plantlet.

**Supplementary Table 1.** Standard curves of hinesol, β-eudesmol, atractylon and atractylodin.

| Volatile oils | Standard curve | R^2^ |
| --- | --- | --- |
| Hinesol | Y = -754757+770768*X | 0.999 |
| β-Eudesmol | Y = -637226+583924*X | 0.999 |
| Atractylon | Y = - 1282820+1483960*X | 0.999 |
| Atractylodin | Y = - 10053200+2111340*X | 0.999 |
